# Early fault detection in gearboxes via dynamic principal component analysis–driven multivariate statistical process control

**DOI:** 10.1371/journal.pone.0348497

**Published:** 2026-05-18

**Authors:** Antonio Pérez-Torres, Jean Navarrete-Campos, Reinier Fernández-López, Jorge Figueroa-Zúñiga, Susana Barceló-Cerdá

**Affiliations:** 1 Department of Applied Statistics and Operational Research, and Quality, Universitat Politècnica de València, València, Spain; 2 Grupo de Investigación y Desarrollo en Tecnologías Industriales (GIDTEC), Universidad Politécnica Salesiana, Cuenca, Ecuador; 3 Departamento de Estadística, Universidad de Concepción, Concepción, Chile; 4 Facultad de Ingeniería y Arquitectura, Universidad Central de Chile, La Serena, Chile; University of Hamburg: Universitat Hamburg, GERMANY

## Abstract

Early detection of gearbox failure is essential due to their critical role in industrial operations. Therefore, effective condition monitoring techniques are required to identify incipient deviations in operational behaviour. Therefore, this study proposes a dynamic principal component analysis methodology, integrated within a multivariate statistical process control framework, to detect progressive failures in spur gearboxes from vibration signals. The signal is segmented into sub-windows and characterised using condition indicators in the time and frequency domains. Diagnosis is based on Hotelling’s *T*^2^ statistic and the squared prediction error, which define statistical control limits to discriminate between normal and failure conditions. Empirical validation uses an experimental dataset covering combinations of load, speed, and failure severity. The results demonstrate high sensitivity to progressive degradation and accurate early-stage detection, supporting the multivariate statistical process control approach with dynamic principal component analysis as an effective tool for diagnosis and predictive maintenance in high-criticality industrial environments.

## 1 Introduction

Gearboxes are essential components in the mechanical power transmission of systems operating across various sectors, including aerospace, automotive, energy, manufacturing, mining, and rail transport, among others [1]. Despite their robust designs, gearboxes are subjected to demanding operating conditions that can lead to progressive failures. For example, wear, cracks, fractures, micropitting, misalignment, contact fatigue, and corrosion [2,3]. Therefore, early detection of these failures is crucial to prevent unplanned shutdowns and ensure the system’s continued operational integrity [4,5].

Among the various condition monitoring (CM, understood as the continuous assessment of operating parameters to detect and diagnose failures) methodologies, vibration analysis stands out for its sensitivity in identifying characteristic patterns generated by mechanical failures, particularly in rotating machinery such as gearboxes [6,7]. Specifically, gearbox signals are often affected by noise and by variable operating conditions (speed-load), which drives the use of increasingly complex and high-dimensional diagnostic approaches [8,9].

However, CM in gearboxes has the drawback that failure signatures are often weak at incipient stages and are modulated by the operating regime (speed-load) and by the inherent dynamics of the transmission system, which can mask subtle changes in the signal. In practice, this leads to datasets with multiple simultaneous, highly correlated indicators and variability induced by operating conditions, which increases the effective dimensionality of the problem and requires multivariate methods capable of separating nominal variation from deviations attributable to failure [6,7,10].

The vibration signal captures the system’s dynamic signature, enabling the extraction of condition indicators (CIs, statistical parameters computed from the signal for feature extraction), which support data-driven diagnosis [11,12]. In addition, advances in data acquisition systems and in multivariate analysis techniques, in particular multivariate statistical process control (MSPC), have established them as practical tools for analysing high-dimensional data [10,13,14].

In this context, MSPC is particularly attractive because it enables the joint monitoring of multiple correlated variables, the establishment of statistical control limits under normal operating conditions (NOC), and the detection of multivariate deviations without requiring labelled failures. This approach is especially pertinent when indicators derived from vibration signals are used, since decisions based on a single variable can be unstable. In contrast, multivariate assessment improves the statistical traceability of changes and reduces ambiguities in the presence of noise [10,15,16].

It is worth noting that principal component analysis (PCA) is a widely used method for dimensionality reduction and for extracting relevant patterns of the system’s operational behaviour [2,6,17,18]. However, conventional PCA assumes temporal independence among observations, limiting its applicability in systems with significant temporal dynamics. This limitation is overcome by dynamic PCA (DPCA), which incorporates time-lagged variables, capturing the correlation between variables and the temporal structure of the process [6,19]. This improvement is particularly beneficial in failure diagnosis for rotating machinery, where failures often develop progressively [20–22].

In particular, in segmented vibration signals, autocorrelation and memory effects arising from system dynamics can shift energy between components and residuals, altering the sensitivity of PCA-based schemes if temporal dependencies are ignored. Therefore, DPCA is relevant for gearbox monitoring, as it introduces a temporal embedding (lags) that allows the sequential structure to be modelled explicitly and thereby improves the detectability of incipient changes within an MSPC framework [6,12,20].

On the other hand, numerous studies have shown that using DPCA significantly improves sensitivity in failure detection for rotating systems, such as wind turbines, cutting tools, and bearings, especially when the temporal structure of the data is incorporated [12,23–25]. For example, Jin et al. [17] showed that an MSPC scheme based on DPCA achieves higher failure detection rates in bearings by explicitly modelling the inherent dynamics of vibration signals. Thus, within the MSPC context, the DPCA-based approach enables quantification of multivariate deviations within the principal subspace and in the model residuals. Statistical control limits are established to define the system’s NOC, and these limits are compared with failure or anomaly scenarios using control charts. Implementing this methodology enables continuous, automated monitoring of critical system states, a vital aspect for maintaining operational reliability [2,7,15,17].

Therefore, this work aims to develop a condition monitoring scheme based on MSPC using DPCA to detect incipient failures in spur gearboxes. As the data source, vibration signals recorded under controlled laboratory conditions are used. The novelty of this work lies not only in integrating dynamic multivariate analysis with statistical control charts, but also in its application to spur gearboxes, for which no precedents have been reported in the literature. This contribution positions the study as a bridge between vibration-based engineering diagnosis and data-driven statistical process control methods, as discussed in [16,26].

Finally, the remainder of the article is organised as follows. Section 2 details the MSPC methodology based on DPCA, emphasising the separation between Phase I and Phase II and the cross-validation-based selection of lags and components. Section 3 describes the test rig and the data. Section 4 presents the empirical results, including the Phase I control limits, the analysis of detection delay across different severity levels in Phase II, and a discussion of why the *SPE* chart achieves earlier detection when the correlation structure breaks down. Finally, Section 5 summarises the main findings and outlines directions for future work.

## 2 Methodology

DPCA extends standard PCA to model industrial processes with temporal autocorrelation [27,28]. Unlike standard PCA, which assumes independence between consecutive observations, DPCA captures dynamic dependencies arising from transient states or memory effects, which are common in physical systems and industrial control environments [29,30]. A defining feature of DPCA is the explicit incorporation of time lags into the data matrix, which enables effective modelling of the sequential structure inherent to multivariate systems [31,32]. This capability is particularly valuable for monitoring mechanical processes, where gradual or smooth transitions contain relevant information about the evolution of the system’s operating state [27,33]. Therefore, each observation is represented as a concatenation of the original series and its time-lagged versions:


$${𝐅}_{t}={\left[\begin{array}{llll} {𝐟}_{t}^{⊤} & {𝐟}_{t-1}^{⊤} & \cdots & {𝐟}_{t-p}^{⊤} \end{array}\right]}^{⊤},\;{𝐅}_{t}\in {\mathbb{R}}^{m(p+1)},$$ (1)

where ${𝐟}_{t}\in {\mathbb{R}}^{m}$ is the vector of CIs at time *t*, *p* denotes the number of lags considered, and *m* is the number of variables. S*t*acking these lag-augmented observations by rows yields the DPCA data matrix [34]:


$${𝐗}_{D}=[\begin{array}{l} {𝐅}_{1}^{⊤}\\ {𝐅}_{2}^{⊤}\\ \vdots \\ {𝐅}_{n}^{⊤} \end{array}]\in {\mathbb{R}}^{n\times m(p+1)},$$ (2)

where *n* is the number of observations available after accounting for the lags, Takens’ theorem [35] justifies reconstructing the state space of dynamical systems from time-lagged observations.

The DPCA model is estimated via singular value decomposition (SVD) or the spectral decomposition of ${𝐗}_{D}^{⊤}{𝐗}_{D}$ [36], which leads to:


$$𝐓={𝐗}_{D}\;{𝐏}_{k},\;{𝐏}_{k}=[{𝐩}_{1},\ldots ,{𝐩}_{k}],$$ (3)

where **P**_*k*_ contains the first *k* eigenvectors (loadings) and $𝐓\in {\mathbb{R}}^{n\times k}$ are the corresponding scores.

Before fitting the model, the variables are centred and scaled using Phase I statistics, with $\mu_{P_{0}}\in {\mathbb{R}}^{m}$ and $\sigma_{P_{0}}\in {\mathbb{R}}^{m}$ denoting the vectors of means and standard deviations estimated from NOC data. That is, with the gearbox in a healthy state, denoted as *P*_0_ (see Table 1). Each observation is standardised as:


$${𝐟}_{t}^{\prime }=({𝐟}_{t}-\mu_{P_{0}})⊘\sigma_{P_{0}},$$ (4)

where ⊘ denotes element-wise division.

**Table 1:** Severity of pinion tooth-break failure.

Severity	Failure description	Tooth loss
*P*_0_	Healthy condition	0.00%
*P*_1_	Failure volume of 4.64 mm^3^ on one tooth	1.30%
*P*_2_	Failure volume of 14.29 mm^3^ on one tooth	4.00%
*P*_3_	Failure volume of 26.79 mm^3^ on one tooth	7.50%
*P*_4_	Failure volume of 40.36 mm^3^ on one tooth	11.30%
*P*_5_	Failure volume of 72.87 mm^3^ on one tooth	20.40%
*P*_6_	Failure volume of 109.30 mm^3^ on one tooth	30.60%
*P*_7_	Failure volume of 145.74 mm^3^ on one tooth	40.80%
*P*_8_	Failure volume of 250.75 mm^3^ on one tooth	70.20%
*P*_9_	Failure volume of 357.20 mm^3^ on one tooth	100.00%

In Phase II, new observations are normalised using these same frozen *P*_0_ parameters, ensuring consistency between phases and preventing information leakage.

The hyperparameters (*k*,*p*) are selected using a cross-validation (CV) procedure that minimises the Squared Prediction Error (*SPE*) while maintaining a stable in-control Average Run Length (*ARL*_0_) [37–40]. This is defined as:


$$\begin{array}{l} \begin{array}{l} \begin{array}{ll} score & =\mathrm{median}\;\left(\frac{{SPE}_{\text{test}}}{{UCL}_{SPE}}\right),\\ {RL}_{r} & =\left\{ \begin{array}{ll} \min\left\{\;t\in \{1,\ldots ,N_{r}\}:\;{SPE}_{r,t}>{UCL}_{SPE}\right\}, & \text{if the set is nonempty},\\ N_{r}, & \text{otherwise}, \end{array}\right.\\ {ARL}_{0} & ={\mathrm{median}}_{r}\;\left({RL}_{r}\right),\\ score & \leftarrow score\cdot \left\{ \begin{array}{ll} 1-\frac{{\min(ARL}_{0})-{ARL}_{0}}{{\min(ARL}_{0})}, & \text{if }{ARL}_{0}<{\min(ARL}_{0}),\\ 1, & \text{otherwise}. \end{array}\right. \end{array} \end{array} \end{array}$$ (5)

where *RL*_*r*_ is the run length of run *r* (the first time instant or segment at which the *SPE* statistic crosses the upper control limit), *N*_*r*_ is the total number of segments in run *r*, and the score is a performance index.

This scheme uses time-block partitions to prevent temporal leakage and estimates *ARL*_0_ under NOC via simulation or resampling of standardised sequences. In this way, the resulting model balances predictive capability with the stability required for statistical monitoring. As a reference, the classical variance-retention criterion is defined as:


$$k=\min\left\{j\;|\;\frac{\sum_{i=1}^{j}\lambda_{i}}{\sum_{i=1}^{a}\lambda_{i}}\geq \gamma \right\},$$ (6)

with *a* = *m*(*p* + 1) and a threshold γ that in practice typically lies in the range 0.70–0.90 [23,41]. However, this threshold is replaced by a CV-based optimisation, providing a more robust selection of model complexity.

On the other hand, within the MSPC framework, the DPCA model operates in two complementary phases. In Phase I, NOC are established, **P**_*k*_ and the diagonal matrix $\Lambda =diag(\lambda_{1},\ldots ,\lambda_{k})$ of retained eigenvalues are fixed, and the control limits at significance level α are computed. In Phase II, new observations with possible failures are standardised using the NOC parameters, projected onto the frozen **P**_*k*_, and the Hotelling *T*^2^ and *SPE* statistics are evaluated.

Hotelling’s *T*^2^ statistic is defined as:


$$T^{2}(t)={𝐭}_{t}^{⊤}\Lambda^{-1}{𝐭}_{t}$$ (7)

where **t**_*t*_ is the score vector at time *t*. The *SPE* is given by:


$$SPE(t)=\|{𝐅}_{t}-{\hat{𝐅}}_{t}{\|}^{2}=\|{𝐅}_{t}-{𝐏}_{k}{𝐏}_{k}^{⊤}{𝐅}_{t}{\|}^{2},$$ (8)

where ${𝐅}_{t}$ is the observation at time *t* and ${\hat{𝐅}}_{t}$ is its projection onto *t*he principal subspace. The first statistic evaluates the multivariate distance within the principal subspace, whereas the second quantifies the residual variance not explained by the model. Upper Control Limits (*UCL*) are defined for both statistics. For *T*^2^, the *UCL* is obtained from the Snedecor *F* distribution with *n*_0_ effective Phase I observations (after accounting for *p* lags):


$${UCL}_{T^{2}}=\frac{k(n_{0}-1)}{n_{0}-k}\;F_{1-\alpha }(k,n_{0}-k).$$ (9)

In contrast, the *UCL* for *SPE* uses the Jackson–Mudholkar approximation [42]. To this end, the total number of variables *a* = *m*(*p* + 1) after temporal expansion is considered, and moments of the residual eigenvalues, which reflect the variability not explained by the model, are computed as $\theta_{i}=\sum_{j=k+1}^{a}\lambda_{j}^{\;i}$ for *i* = 1,2,3, with $h_{0}=1-\frac{2\theta_{1}\theta_{3}}{3\theta_{2}^{2}}$. Thus,


$${UCL}_{SPE}=\theta_{1}{\left(1+\frac{z_{1-\alpha }\sqrt{2\theta_{2}}\;h_{0}}{\theta_{1}}+\frac{\theta_{2}\;h_{0}\;(h_{0}-1)}{\theta_{1}^{2}}\right)}^{1/h_{0}}.$$ (10)

These thresholds define safe operating regions and enable automatic alarm triggering in response to significant deviations, thereby enhancing the capability for effective real-time monitoring [7,15].

As part of the monitoring protocol, Phase I fits the DPCA model and selects (*p*,*k*) via block CV to minimise *SPE* subject to an in-control *ARL*_0_ constraint. The pair of limits $\left({UCL}_{T^{2}},{UCL}_{SPE}\right)$ is computed at significance level α, and the set $\left\{\mu_{P_{0}},\sigma_{P_{0}},{𝐏}_{k},\Lambda ,{UCL}_{T^{2}},{UCL}_{SPE}\right\}$ is frozen. In Phase II, each new observation is standardised using the *P*_0_ values, dynamically embedded using the frozen *p*, projected onto the fixed subspace, and *T*^2^(*t*) and *SPE*(*t*) are evaluated against their respective *UCL*s. For slow degradations, memory charts, Exponentially Weighted Moving Average (EWMA) or Cumulative Sum (CUSUM), are considered, applied to *SPE* or *T*^2^, with parameters (λ,k,h) tuned via CV [37,38].

To detect gradual or low-magnitude degradations, memory charts are applied to the *T*^2^ or *SPE* statistics. For example, the EWMA chart updates its cumulative value as:


$$Z_{t}=\lambda \;S(t)+(1-\lambda )\;Z_{t-1},\;0<\lambda \leq 1,$$

where $S(t)\in \{T^{2}(t),SPE(t)\}$ is the instantaneous statistic and λ controls the weight of the memory (smaller values increase sensitivity to slow changes). An alarm is triggered when *Z*_*t*_ exceeds its upper control limit ${UCL}_{EWMA}$.

Whereas the CUSUM chart accumulates successive deviations from the expected mean:


$$C_{t}=\max(0,\;C_{t-1}+S(t)-k),$$

where *k* is the reference value and *h* is the decision threshold; an alarm is triggered when $C_{t}>h$. Both configurations (λ,k,h) are optimised via block CV in Phase I, ensuring an in-control *ARL*_0_ consistent with significance level α. Operational details are summarised in Algorithms B2 and B3.

**Algorithm 1. Phase I: calibration and freezing.**

**Require:** Dataset *P*_0_; grid 𝒫×𝒦 for (*p*,*k*); significance level α; target *ARL*_0_; time-blocked CV scheme

1: Estimate $\mu_{P_{0}},\sigma_{P_{0}}$ on *P*_0_; standardize *P*_0_ with these parameters

2: **for all** (p,k)∈𝒫×𝒦 **do**

3:    Define time-blocked folds to avoid leakage

4:    **for all** folds **do**

5:      Fit DPCA on training blocks $\Rightarrow {𝐏}_{k}$, Λ

6:      Compute *SPE* on validation blocks

7:    **end for**

8:    SPE(p,k)← average validation *SPE* across folds

9:    Estimate *ARL*_0_(*p*,*k*) under NOC via simulation/resampling at level α

10: **end for**

11: Select $\left(p^{*},k^{*})=\arg\min\;{SPE}_{OOF}(p,k\right)$ subject to $ARL_{0}(p,k)\geq ARL_{0}^{target}$

12: Refit DPCA on full standardized *P*_0_ with $\left(p^{*},k^{*}\right)$ to obtain ${𝐏}_{k^{*}}$ and Λ

13: Compute ${UCL}_{T^{2}}$ and *UCL_SPE_* at level α using the effective sample size *n*_0_

14: **Freeze and store** $\left\{\mu_{P_{0}},\sigma_{P_{0}},p^{*},{𝐏}_{k^{*}},\Lambda ,{UCL}_{T^{2}},{UCL}_{SPE}\right\}$

15: **return** Frozen parameter set for Phase II

**Algorithm 2. Phase II: monitoring workflow.**

**Require:** Frozen $\left\{\mu_{P_{0}},\sigma_{P_{0}},p^{*},{𝐏}_{k^{*}},\Lambda ,{UCL}_{T^{2}},{UCL}_{SPE}\right\}$

1: **(Warm-up)** If $t<p^{*}$, **skip** evaluation or start at $t=p^{*}$

2: ${𝐟}_{t}\leftarrow ({𝐟}_{t}-\mu_{P_{0}})/\sigma_{P_{0}}$ ▷ Standardize with *P*_0_ (no re-estimation)

3: ${𝐅}_{t}\leftarrow [{𝐟}_{t}^{⊤},{𝐟}_{t-1}^{⊤},\ldots ,{𝐟}_{t-p^{*}}^{⊤}{]}^{⊤}$ ▷ Dynamic embedding with frozen $p^{*}$

4: ${𝐭}_{t}\leftarrow {𝐏}_{k^{*}}^{⊤}{𝐅}_{t}$ ▷ Fixed projection with frozen loadings

5: $T^{2}(t)\leftarrow {𝐭}_{t}^{⊤}\Lambda^{-1}{𝐭}_{t}$

6: $SPE(t)\leftarrow \|{𝐅}_{t}-{𝐏}_{k^{*}}{𝐏}_{k^{*}}^{⊤}{𝐅}_{t}{\|}^{2}$

7: $A_{T^{2}}\leftarrow True$ if $T^{2}(t)>{UCL}_{T^{2}}$ else False

8: $A_{SPE}\leftarrow True$ if *SPE*(*t*)>*UCL_SPE_* else False

9: **Optional memory charts:** update EWMA/CUSUM on *T*^2^ and/or SPE for slow drifts

10: **return** $\left(A_{T^{2}},A_{SPE}\right)$

Regarding the model evaluation metrics and the definition of detection delay, let *t*_0_ denote the start of the failure segment on the segment scale (see Section 3). For each control statistic $S(t)\in \{T^{2}(t),\;SPE(t)\}$, the detection delay is defined as the number of segments elapsed from failure onset to the first crossing of the upper control limit:


$$d_{S}\;=\;\min\{\;t\geq t_{0}:S(t)>{UCL}_{S}\;\}\;-\;t_{0},$$

where *d*_*S*_ is measured in segments. In particular, $d_{T^{2}}$ and *dSPE* denote the detection delays obtained with the *T*^2^ and *SPE* charts, respectively. We also report the medians and interquartile ranges (IQR) of these values, grouped by failure severity level, where $IQR(d_{S})=Q_{0.75}(d_{S})-Q_{0.25}(d_{S})$. In addition, we quantify three complementary metrics:

(i) *ARL*_0_ during Phase I,
(ii) the percentage of out-of-control observations (%OOC) for each statistic, and
(iii) monotonicity with failure severity via Spearman’s rank correlation coefficient $\hat{\rho }$.

These are estimated, along with confidence intervals, using bootstrap resampling. The %OOC quantifies the fraction of segments that exceed the *UCL* of a given statistic:


$$\%{OOC}_{S}\;=\;100\times \frac{1}{N}\sum_{t=1}^{N}𝕀(S(t)>{UCL}_{S}),\;S\in \{T^{2},SPE\},$$

where *N* is the total number of segments evaluated and 𝕀(·) is the indicator function, which takes the value 1 when the statistic *S*(*t*) lies above the upper control limit and 0 o*t*herwise. Consequently, this function acts as a counter that records the number of out-of-control segments. Under *P*_0_, we expect %OOC≈100α%, whereas increases in Phase II reflect deviations from NOC.

It is worth noting that the use of the Snedecor *F* distribution to approximate the control limit for Hotelling’s *T*^2^ statistic is grounded in classical MSPC developments. In particular, Montgomery [43] shows that under multivariate normality, independence between observations, and estimation of the covariance matrix in Phase I, the *T*^2^ statistic can be transformed to follow approximately an *F* distribution [30].

This approximation provides an operational reference for defining control limits in MSPC schemes. However, it has limitations when the stated assumptions are not strictly satisfied. For example, in scenarios with small sample sizes, pronounced serial dependence, or covariance matrices estimated from limited Phase I information, the *T*^2^ statistic may deviate from the *F* distribution, affecting the calibration of the control limit [22,44]. Therefore, biases may arise in the false-alarm rate (inflation or deflation of *ARL*_0_) and, in extreme contexts, sensitivity to incipient failures may be reduced.

This phenomenon has been documented in recent studies on monitoring vibration systems and dynamic processes, where dimensionality and temporal dependence influence the empirical distribution of control statistics [7,23]. To mitigate these limitations, our study uses a rigorous Phase I calibration procedure based on time-block CV and *ARL*_0_ simulation, which ensures that the ${UCL}_{T^{2}}$ employed is empirically matched to the actual data structure under NOC. In this way, any potential deviation from the *F* distribution is absorbed into the experimental estimation of *ARL*_0_, ensuring a consistent false-alarm rate and stability of the monitoring system.

All of the above ensures that the model remains fixed during Phase II; new data are only projected and evaluated against the objective limits defined in Phase I [16,26]. The resulting DPCA–MSPC framework ensures statistical consistency between phases and provides a solid basis for the performance analysis discussed in Section 4.

**Fig 1:**
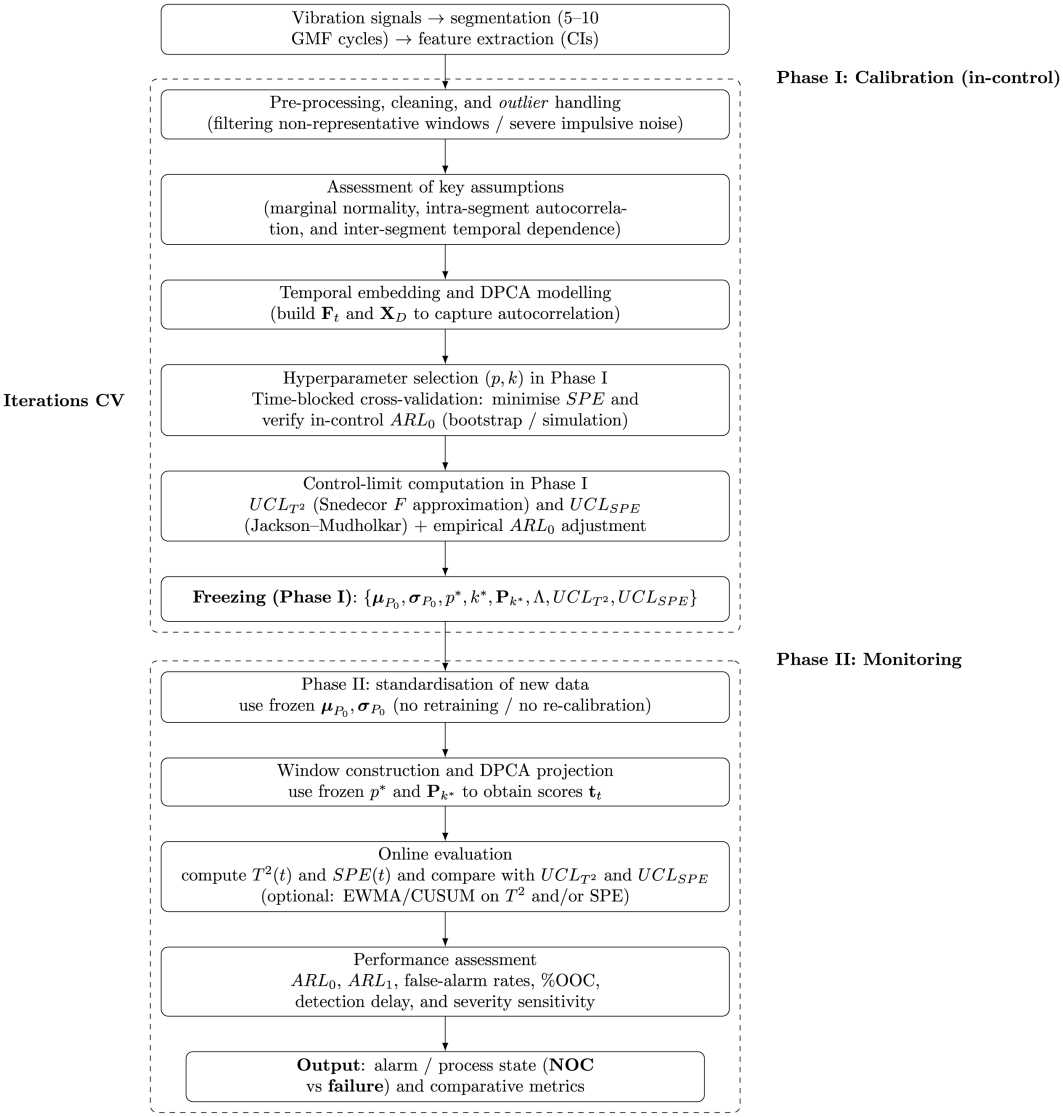
Workflow of the proposed DPCA–MSPC scheme, distinguishing Phase I (calibration and freezing) and Phase II (monitoring).

Next, we detail the two-phase framework, following SPC and multivariate monitoring recommendations for dynamic systems [30,45,46]. This framework is depicted in the flowchart shown in Fig 1.

**Phase I:** Establishing the in-control model. In this stage, we follow a rigorous methodological sequence to ensure that the DPCA–MSPC model is built from data representative of the healthy state. The procedure is as follows:

1. Pre-processing, cleaning, and outlier handling. We implement initial filtering to remove windows that are unrepresentative or affected by extreme impulsive noise, in accordance with the condition indicators’ consistency criteria.
2. Assessment of fundamental assumptions. We analyse marginal normality, within-segment autocorrelation, and temporal dependence between segments.
3. Transformations and appropriate modelling. Because autocorrelation is present, we apply the DPCA extension to capture the temporal structure, replacing the need for ad hoc transformations and providing a more suitable model for vibration signals.
4. In-control model estimation and optimal selection of hyperparameters (*p*, *k*). We implement a time-block CV, minimise *SPE*, and verify *ARL*_0_ via bootstrap simulation.
5. Computation of *T*^2^ and *SPE* control limits with empirical *ARL*_0_ adjustment. We verify control-limit calibration by integrating the classical *F* approximation with an empirical bootstrap estimate to make *ARL*_0_ more robust.

**Phase II:** Process monitoring. Once the in-control model is fixed, we proceed with strict monitoring under standard multivariate SPC rules:

1. Standardising new data using frozen Phase I parameters. We do not recalibrate any parameters; this avoids contamination of failure information.
2. Constructing time windows and DPCA projection. Each window preserves the temporal structure defined in Phase I, ensuring direct comparability.
3. Online evaluation using *T*^2^ and *SPE*. We apply the *UCL* limits computed in Phase I. Optional memory charts (EWMA, CUSUM) optimised via CV are also considered.
4. Performance comparison: *ARL*_0_, *ARL*_1_, false-alarm rates, and detection capability. We add comparative metrics that allow the robustness and sensitivity of the monitoring system to be assessed.

To improve the clarity, transparency, and reproducibility of the proposed procedure, Appendix B presents a complete step-by-step workflow based on the real experimental data obtained from the test bench described in Section 3.

## 3 Test bench and data

**Fig 2:**
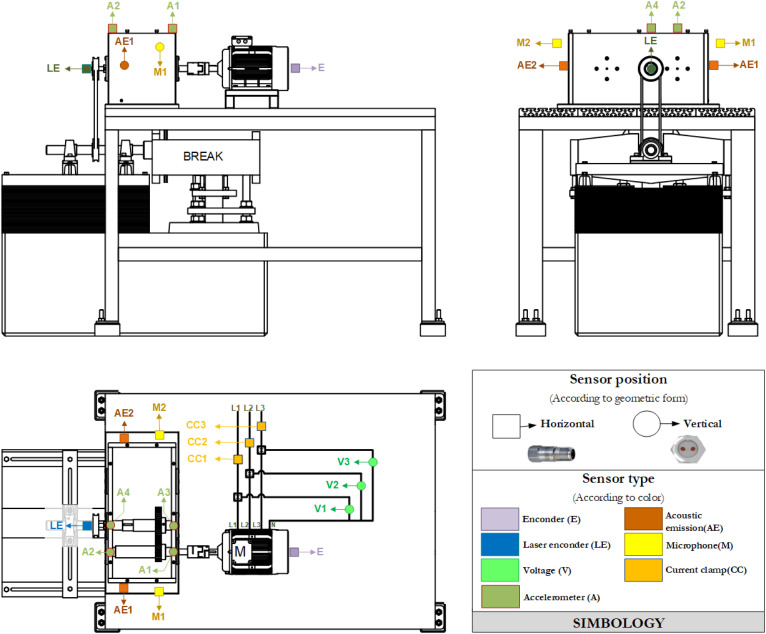
Test bench layout.

The test bench used in the experimental phase (Fig 2) consists of a single-stage spur gearbox coupled to a three-phase motor rated at 2 HP, 220 V, and 1,200 rpm. We controlled the motor speed using a variable-frequency drive, enabling simulation of different speed conditions. We integrated an electromagnetic brake on the output shaft to apply different mechanical loads to the test bench.

The gearbox comprises a pinion, *Z*_1_, with 32 teeth and a gear, *Z*_2_, with 48 teeth. To simulate different mechanical degradation scenarios, we deliberately introduced ten severity levels of pinion tooth-break failure, labelled from *P*_0_ (healthy condition) to *P*_9_ (severe failure), as detailed in Table 1. We evaluated severity levels for specific combinations of rotational speeds (8 Hz, 14 Hz, and 20 Hz) using a variable-frequency drive. We applied load levels using the electromagnetic brake (0 V, 10 V, and 20 V) and replicated each experimental configuration 10 times with 10-second tests. In total, we obtained 900 records per sensor, each with 500,000 acceleration samples (measured in *m*/*s*^2^), providing a robust dataset for statistical analysis and diagnostic model validation.

**Fig 3:**
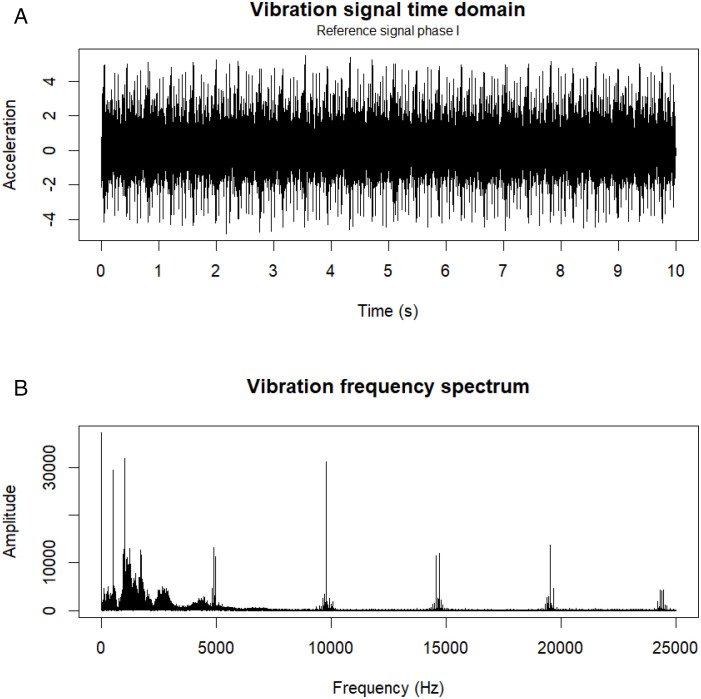
Vibration signal.

We acquired vibration data in the time domain (Fig 3a) using four vertically mounted accelerometers (*A*_1_–*A*_4_), and we subsequently transformed this signal to the frequency domain (Fig 3b) using the fast Fourier transform (FFT). Sensors *A*_1_ and *A*_2_ were installed on the input shaft, whereas *A*_3_ and *A*_4_ were located on the gearbox output shaft (the test bench also included acoustic emission, voltage, current-clamp, microphone, encoder, and laser encoder sensors). The sampling frequency of each channel was 50 kHz, providing high temporal resolution for dynamic analysis. We designed this experimental configuration to capture both the direct excitation generated by failures in the input gear teeth and the dynamic response propagated along the test bench. Defects in spur gears produce distinctive vibration signatures, which are influenced by the failure and by the prevailing operating conditions [47,48].

(a) Time domain
(b) Frequency domain

Table 2 provides descriptive information for the original vibration signal in the time domain with the pinion in healthy condition *P*_0_ (Phase I) and the nine severity levels *P*_1_–*P*_9_ (Phase II).

**Table 2:** Descriptive statistics of the original vibration signal in the time domain.

Type	Severity	Mean	Median	SD	Min	Max	Q_05_	Q_25_	Q_75_	Q_95_
Reference (Phase I)	*P*_0_	0.07	0.06	0.76	−4.79	5.53	−1.09	−0.36	0.49	1.30
Fault (Phase II)	*P*_1_	0.07	0.08	0.48	−2.99	3.26	−0.69	−0.20	0.34	0.79
Fault (Phase II)	*P*_2_	0.06	0.06	1.63	−9.43	10.35	−2.55	−0.78	0.87	2.71
Fault (Phase II)	*P*_3_	0.07	0.06	0.83	−6.15	5.59	−1.25	−0.41	0.54	1.39
Fault (Phase II)	*P*_4_	0.08	0.11	1.96	−12.28	12.70	−2.99	−0.87	0.97	3.17
Fault (Phase II)	*P*_5_	0.07	0.15	2.33	−9.22	10.74	−3.76	−1.53	1.54	3.86
Fault (Phase II)	*P*_6_	0.06	0.02	1.23	−6.75	6.98	−1.83	−0.74	0.81	2.13
Fault (Phase II)	*P*_7_	0.06	0.10	0.80	−5.55	5.55	−1.23	−0.42	0.57	1.24
Fault (Phase II)	*P*_8_	0.07	0.13	1.02	−4.97	4.61	−1.79	−0.48	0.70	1.60
Fault (Phase II)	*P*_9_	0.07	0.12	0.84	−7.87	8.39	−1.31	−0.33	0.53	1.17

The vibration signal is first divided into consecutive, non-overlapping time windows, so that each window captures a local portion of the process dynamics and can be treated as an individual observation for monitoring. Under this criterion, we define a segment as a time window containing between five and ten complete cycles of the Gear Mesh Frequency (GMF), with


$$GMF=f_{r}\times Z_{1},$$

where *f*_*r*_ is the rotational frequency of the pinion *Z*_1_, this segmentation strategy allows the temporal evolution of the vibration signal to be represented through successive local observations, while preserving sufficient information to detect incipient changes associated with fault development. Moreover, this range (5–10 GMF cycles) balances spectral resolution and sensitivity to transients [49,50]. We compute the *T*^2^ and *SPE* statistics per segment and measure detection delays as the number of segments.

After segmentation, each time window is characterised by means of a set of condition indicators (CIs) extracted from the corresponding portion of the signal, thereby transforming the raw vibration signal into a sequence of multivariate observations suitable for statistical monitoring. An iterative procedure involving resampling and parameter tuning determined the optimal number of sub-windows per signal, thereby maximising sensitivity to incipient faults and dynamic fluctuations. We extracted 10 CIs from each time sub-window and computed them in both the time and frequency domains.

In the time domain, we used the following CIs: mean, standard deviation, kurtosis, skewness, shape factor, impulse factor, clearance factor, crest factor, zero crossings, and higher-order time moments. In the frequency domain, we considered the following signal features: skewness, kurtosis, centre frequency, standard deviation, root mean square, relative dispersion ratio, shape indicator, second spectral moment, third spectral moment, and fourth spectral moment. The mathematical formulations of these CIs are presented in detail in Table 7 (Appendix A).

## 4 Results and discussion

This section evaluates the performance of the DPCA–MSPC scheme following the methodological workflow described in Section 2, which explicitly distinguishes between the calibration phase (Phase I) and the monitoring phase (Phase II). In Phase I, we select the hyperparameters (*p*,*k*) via CV, estimate the loadings **P**_*k*_ and the spectrum Λ, and set the upper control limits ${UCL}_{T^{2}}$ and *UCL_SPE_* at significance level α. Subsequently, in Phase II, observations associated with progressive failures are standardised using the frozen Phase I parameters, projected onto the DPCA subspace fixed in Phase I, and evaluated solely against those limits, without retraining or threshold readjustment. Under this protocol, we quantify:

(i) the detection delay by severity (median and IQR);
(ii) the monotonic relationship between severity and post-onset maxima (Spearman $\hat{\rho }$);
(iii) out-of-control rates and alarm triggering; and
(iv) the temporal evolution of *T*^2^ and *SPE*.

The performance metrics are computed at the segment level, defined as a time sub-window containing between 5 and 10 complete cycles of the Gear Mesh Frequency (GMF), as specified in Section 3. Comparability between Phase I and Phase II is ensured because both *T*^2^ and *SPE* are evaluated on homogeneous decision units, and the detection delay is defined as the number of segments from failure onset to the first statistical alarm.

We calibrated the Phase I model on *P*_0_ using time-block CV to select (*p*,*k*) by minimising *SPE* while preserving a stable in-control Average Run Length (*ARL*_0_). The selected configuration was *p* = 0 and *k* = 8 with α=0.01, yielding a median *ARL*_0_ ≈ 36 segments. We computed the upper control limits ${UCL}_{T^{2}}$ and *UCL_SPE_* using the Snedecor *F* approximation and the Jackson–Mudholkar formula, respectively, and then froze them for the whole of Phase II (Table 3). Freezing implies that (*p*,*k*), the loadings **P**_*k*_, and the control thresholds are not recalibrated in the presence of failure.

**Table 3:** Phase I: frozen control limits and model settings.

Method	*p*	*k*	α	*UCL*_*T*2_	*UCL*_*SPE*_	*ARL*_0_ median
DPCA (CV-Selected)	0	5	0.01	15.09	9.99	85
						
DPCA (*ARL*_0_-constrained)	0	8	0.01	23.23	0.76	36
						
DPCA (Phase I optimal)	1	7	0.01	18.49	16.65	97

On the other hand, the results in Table 3 highlight the trade-off between in-control stability (*ARL*_0_) and failure sensitivity. Configurations with higher *ARL*_0_, such as the DPCA model (*p* = 1), reduce the probability of false alarms. However, they can delay the detection of incipient deviations. In contrast, the calibrated specification with *ARL*_0_ = 36 prioritises earlier detection, particularly through the *SPE* statistic, whose significantly tighter threshold increases the ability to identify subtle changes in the process residual structure. This trade-off is relevant in industrial monitoring contexts, where the target *ARL*_0_ depends on system criticality, the costs associated with false alarms, and the operational risk of late detections. Considering both conservative and sensitive configurations enables us to robustly evaluate the DPCA scheme’s Phase II performance across different risk profiles. Notably, the model selected via cross-validation focuses on reconstruction performance and does not necessarily coincide with the optimal configuration for process monitoring. In contrast, the Phase I optimal model is defined in terms of in-control performance, maximising *ARL*_0_ and reducing false alarm rates. This distinction highlights the need to decouple model selection criteria for prediction and monitoring tasks.

In Phase II under failure conditions (*P*_1_–*P*_9_), we standardise each new observation exclusively using the Phase I parameters ($\mu_{P_{0}}$ and $\sigma_{P_{0}}$), represent it as a dynamic vector **F**_*t*_ using the *p* selected in Phase I, and project it onto the frozen loadings **P**_*k*_. We then compute *T*^2^(*t*) and *SPE*(*t*) and compare them with the limits ${UCL}_{T^{2}}$ and *UCL_SPE_* obtained in Phase I (see Algorithm B3). In this scheme, any threshold crossing in Phase II is interpreted directly as a deviation from the NOC defined in Phase I, without requiring model retraining or control-limit readjus*t*men*t*.

Table 4 presents the detection system performance by failure severity and by statistic. Specifically, the *SPE* statistic yields lower median detection delays than *T*^2^ in most scenarios, particularly for higher-severity failures. This pattern suggests that *SPE* is more sensitive to structural changes in the process residual variability. For severities *P*_2_ and *P*_4_, *SPE* shows notably low median delays, with median *SPE* delays between 5 and 6 segments, accompanied by low *ARL*_*SPE*_ values, indicating rapid failure detection. In particular, case *P*_4_ stands out for low values of $ARL_{T^{2}}=1.43$ and *ARL*_*SPE*_ = 1.69. In addition, *P*_4_ exhibits reduced standard deviations (*SDRL*), reflecting highly stable control-scheme behaviour under severe failures. In contrast, severities *P*_1_, *P*_3_, *P*_5_, and *P*_6_ show higher median delays and considerably larger *ARL* values, especially for the *T*^2^ statistic.

**Table 4:** Detection performance by severity failure. Phase II.

Severity	Median *T*^2^ delay	Median *SPE* delay	*IQR*_*SPE*_	$ARL_{T^{2}}$	$SDRL_{T^{2}}$	$ARL_{SPE}$	*SDRL*_*SPE*_
*P*_1_	24.00	18.00	[5.00, 43.00]	31.78	52.51	13.20	25.65
*P*_2_	22.50	6.00	[3.00, 14.00]	6.39	24.72	7.57	21.26
*P*_3_	21.00	17.00	[4.00, 55.20]	19.01	46.98	23.26	47.97
*P*_4_	28.00	5.00	[2.00, 13.20]	1.43	1.02	1.69	1.63
*P*_5_	20.00	18.00	[3.00, 63.00]	14.30	38.82	16.67	49.33
*P*_6_	26.00	18.50	[5.25, 41.20]	20.46	41.34	20.68	37.25
*P*_7_	21.00	16.50	[4.00, 38.00]	3.59	6.06	4.97	8.59
*P*_8_	14.50	9.00	[4.25, 20.00]	11.03	24.17	24.44	47.82
*P*_9_	20.50	15.00	[7.75, 36.20]	4.43	9.68	3.42	4.43

Accordingly, the associated *SDRL* values are also high, indicating high variability in detection time and, therefore, lower operational reliability for low- to intermediate-severity failures. This pattern is consistent with the statistical process control literature, where incipient failures tend to be more difficult to identify early [45,46]. The interquartile range of *SPE* (*IQR*_*SPE*_) reinforces this interpretation. For high severities, for example, *P*_2_ and *P*_4_ show a narrow *IQR*_*SPE*_, indicating detection concentrated within a few segments. In contrast, for *P*_3_ and *P*_5_, *IQR*_*SPE*_ widens, reaching values above 50 segments, reflecting greater uncertainty in detector performance. It is worth noting that these results confirm that the proposed Phase II scheme is particularly efficient for medium- and high-severity failures. In addition, using the *SPE* statistic improves early detection, particularly for medium- and high-severity failures, although variability may remain high in low-severity cases.

**Fig 4:**
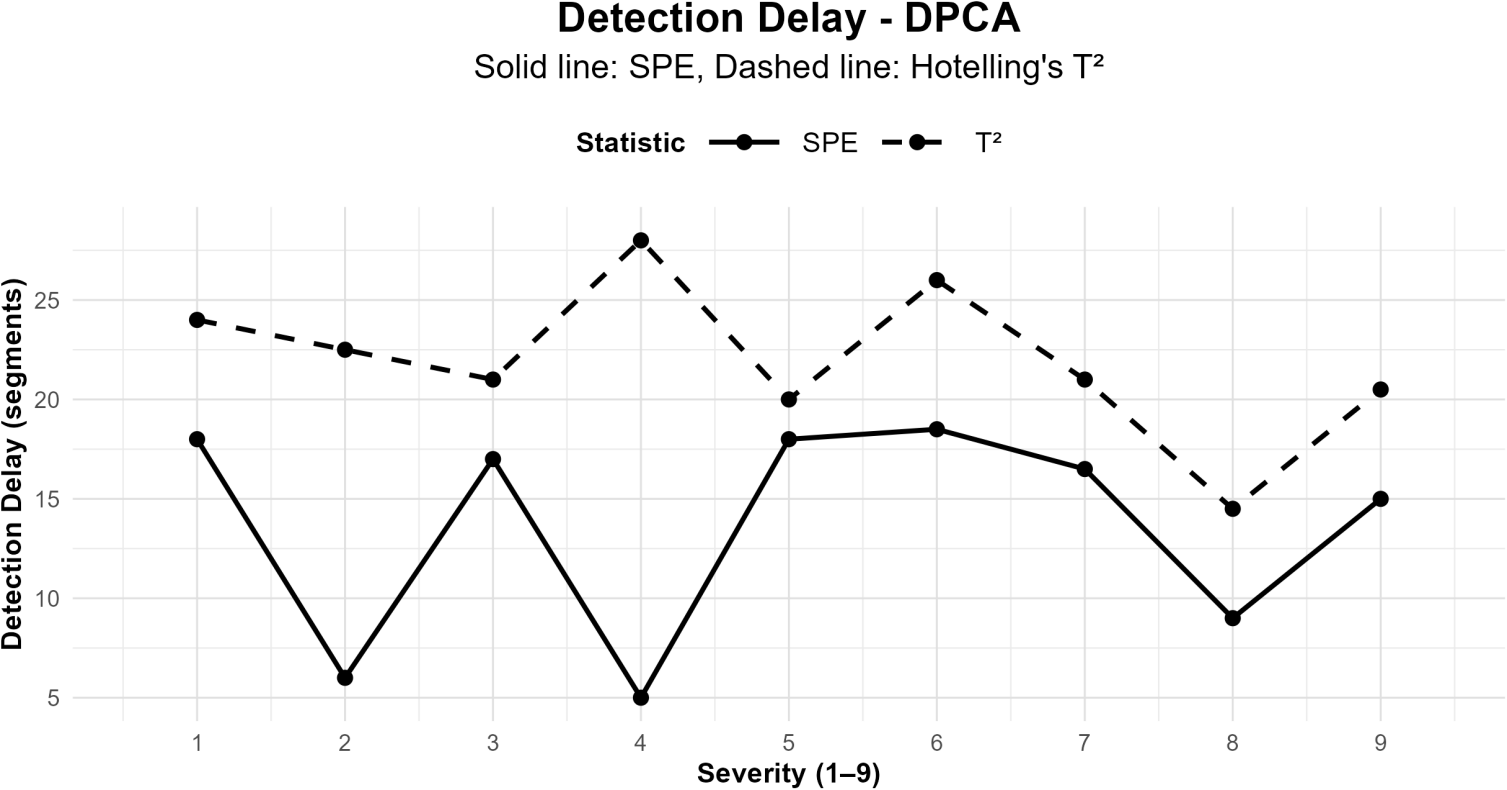
Detection delay by failure severity (Phase II). Solid line: *SPE*; dashed: *T*^2^. The *SPE* chart detects earlier when the correlation structure learnt under NOC is broken.

Fig 4 shows the overall trend across all severities (*P*_1_–*P*_9_): the residual-subspace chart (*SPE*) triggers alarms systematically earlier than *T*^2^ (global medians ${\tilde{d}}_{SPE}\;\approx \;16.5$ versus ${\tilde{d}}_{T^{2}}\;\approx \;21$ segments; see Table 4). This behaviour is consistent with failures that, in the first instance, disrupt the multivariate coherence learned in Phase I, transferring energy towards the residual subspace captured by *SPE* rather than inducing a mean shift within the principal subspace monitored by *T*^2^ [37,38]. The asymmetry between *SPE* and *T*^2^ is also observed qualitatively in the segmented time series (see Figs 6–15 in Appendix C): for most induced severities, *SPE* crosses its frozen Phase I *UCL* (red line) shortly after failure onset, whereas *T*^2^ often remains below its own *UCL* during the first affected segments. To assess whether this advantage of *SPE* increases with severity, we analyse the monotonic (non-parametric) association between failure severity (*P*_1_–*P*_9_) and the post-onset maxima of both control statistics; we use Spearman’s correlation $\hat{\rho }$ with percentile bootstrap confidence intervals.

The results indicate a weak but statistically significant negative association for *SPE* ($\hat{\rho }\approx -0.10$, *p* < 0.01) and a non-significant association for *T*^2^ ($\hat{\rho }\approx 0.02$, *p* = 0.66). We computed these correlations on post-onset maxima, not on detection delays. The trend observed for *SPE* suggests that, as induced severity increases, the residual energy captured by the model also increases, even when multivariate shifts within the principal subspace (*T*^2^) do not exhibit a clear monotonic relationship. This result is consistent with the previously described failure mechanism: early-stage damage initially perturbs the correlation structure learned under NOC, primarily in the residual space (*SPE*) rather than in the principal subspace (*T*^2^).

In addition, we quantified the percentage of segments that exceed the Phase I control limits for each statistic (%*T*^2^ and %*SPE*) and recorded whether alerts were triggered per case (*T*^2^ Alert, *SPE* Alert). Because the limits remain frozen with *k* = 8, variations in out-of-control rates reflect real process changes under fixed thresholds, rather than model readjustment.

Analysis of Table 5 shows that, although the means of *T*^2^ and *SPE* remain relatively stable (around 15 and 8, respectively), the maxima increase with severity (up to 881.34 for *T*^2^ and 361.05 for *SPE*), indicating the presence of localised anomalous episodes captured by the model even when the system’s mean behaviour remains nearly invariant. Consistent with the delay analysis, *SPE* tends to yield higher out-of-control rates than *T*^2^. Alert activation under all evaluated conditions confirms the diagnostic capability of the DPCA–MSPC scheme with Phase I frozen thresholds.

**Table 5:** Statistical results by failure severity.

Severity	Mean *T*^2^	Max *T*^2^	Mean SPE	Max SPE	%*T*^2^	%*SPE*	*T*^2^ Alert	*SPE* Alert
*P*_1_	14.98	283.40	8.19	121.34	13.24	23.51	True	True
*P*_2_	14.98	844.88	8.20	79.07	8.11	33.78	True	True
*P*_3_	14.98	714.60	8.15	198.70	9.34	29.47	True	True
*P*_4_	13.99	510.61	8.26	114.37	5.65	32.96	True	True
*P*_5_	15.98	731.89	8.20	94.13	7.49	34.60	True	True
*P*_6_	16.98	826.67	8.26	361.05	8.62	34.70	True	True
*P*_7_	14.98	720.34	8.82	324.61	14.68	30.90	True	True
*P*_8_	15.98	273.24	8.68	160.31	9.45	37.68	True	True
*P*_9_	14.98	881.34	8.36	90.20	7.49	31.21	True	True

Figs 6–15 in Appendix C illustrate the temporal evolution of *T*^2^ and *SPE* for each failure severity level (*P*_1_–*P*_9_). These segmented trajectories show localised exceedances above the Phase I control limits, which correspond to anomalous episodes in the vibration signals associated with tooth defects. These exceedances appear shortly after failure onset and intensify as severity increases, visually confirming the progressive transition from NOC to increasingly critical states under the same frozen statistical threshold.

On the other hand, Table 6 provides a comparative evaluation of the PCA and DPCA schemes using indicators of in-control stability and signalling capability, considering both the *T*^2^ and *SPE* statistics. The results allow the effects of incorporating temporal dynamics into the DPCA model to be identified. The PCA and DPCA models selected by CV without time lags (*p* = 0) yield identical results across all evaluated metrics. In particular, both methods exhibit an out-of-control (OOC) percentage of 3.62% for *T*^2^ and 1.17% for *SPE*, implying empirical false-alarm rates above the nominal level α=0.01. Likewise, the *SDRL* values associated with *T*^2^ (99.61) and *SPE* (96.97) indicate high dispersion in the signalling delay, reflecting inconsistent temporal stability.

**Table 6:** Comparisons between PCA and DPCA.

Method	%OOC *P*_0_ *T*^2^	%OOC *P*_0_ *SPE*	*SDRL*_*T*2_	p alarm *T*^2^	*SDRL*_*SPE*_	p alarm *SPE*
PCA	3.62	1.17	99.61	0.71	96.97	0.66
DPCA (p = 0, k = 5)	3.62	1.17	99.61	0.71	96.97	0.66
DPCA (p = 1, k = 7)	2.51	2.24	94.60	0.58	97.03	0.63

This result empirically confirms that, in the absence of dynamic structure, DPCA is strictly equivalent to PCA and provides no additional improvement in statistical process control. In contrast, the DPCA specification with temporal dynamics (*p* = 1, *k* = 7) introduces relevant quantitative changes. For the *T*^2^ statistic, the out-of-control percentage decreases from 3.62% to 2.51%, which represents a relative reduction of approximately 31%. This improvement is accompanied by a reduction in *SDRL* from 99.61 to 94.60 (≈5%), as well as a drop in the alarm probability (p alarm) from 0.71 to 0.58. These results indicate that incorporating time-lag filters helps account for serial variability, stabilises the behaviour of the principal subspace, and reduces the frequency and dispersion of false alarms associated with *T*^2^.

Regarding the *SPE* statistic, the out-of-control percentage increases from 1.17% to 2.24%, nearly doubling the empirical signalling rate. This increase is accompanied by an almost unchanged *SDRL* (97.03) and by a slight reduction in p alarm (from 0.66 to 0.63). These results suggest that the temporal dynamics captured by DPCA redistribute process variance, shifting part of the sensitivity towards the residual subspace, where *SPE* becomes more reactive to short-duration deviations or structural changes not explained by the dynamic principal components.

**Fig 5:**
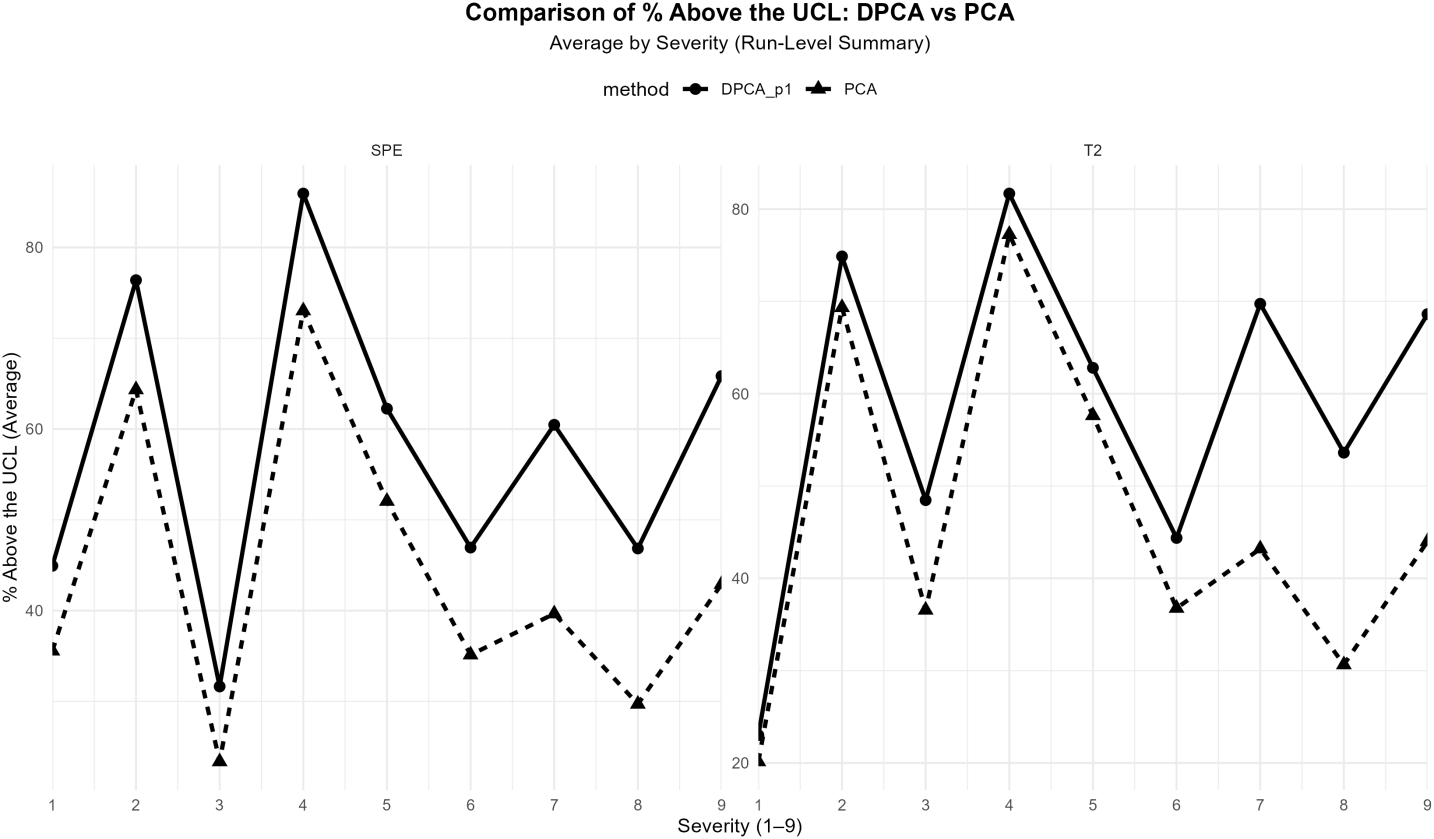
Comparison DPCA vs PCA.

Fig 5 reveals differences when comparing PCA with dynamic DPCA (*p* = 1). For example, dynamic DPCA exhibits systematically higher values of the *SPE* statistic in terms of % above the *UCL* across virtually all severity levels. This separation is particularly marked at intermediate and high severities, where DPCA reaches signalling peaks that far exceed those observed under PCA. This result indicates that incorporating temporal dynamics increases the sensitivity of *SPE* to persistent residual deviations, consistent with the higher out-of-control percentage observed in Table 6. By contrast, the *T*^2^ statistic shows a more moderate behaviour. It is worth noting that both methods (PCA and dynamic DPCA) show increases in % above the *UCL* as severity increases. However, dynamic DPCA tends to generate smoother profiles and, at several levels, values that are comparable to or even lower than those of PCA. This result is consistent with the reduction in OOC percentage and p alarm reported for *T*^2^ in Table 6. It suggests that temporal dynamics help absorb serial dependence within the principal subspace, reducing spurious activation of the *T*^2^ statistic. Therefore, using dynamic DPCA is justified in processes with relevant temporal dependence, where anomalies may manifest gradually and differently across subspaces.

Overall, the results show that integrating DPCA within MSPC enables early and accurate detection of deviations from NOC in spur gearboxes through vibration analysis. The proposed model exhibits high sensitivity to subtle changes in system behaviour, reflected in significant increases in Hotelling’s *T*^2^ and *SPE*, even at early failure stages.

Although a direct comparison with most previous DPCA studies is not straightforward, as they mainly focus on other types of rotating machinery, the empirical evidence supports the approach’s effectiveness. For example, several works report that using DPCA significantly improves failure detection capability in wind turbines, cutting tools, and bearings, particularly by capturing the temporal structure of the signals [12,23–25].

Likewise, the results of this study are consistent with those reported by Baydar et al. [51], who, using singular value decomposition techniques applied to helical gears, identified incipient failures through *SPE* analysis, even without using Hotelling’s *T*^2^. These results reinforce the diagnostic value of multivariate PCA-based approaches for non-invasive characterisation of conditions.

In addition, Jin et al. [17] propose a variant of MSPC for fault-agnostic scenarios, integrating hierarchical clustering (HCA) to improve detection under non-Gaussian conditions. Their proposal highlights the need for adaptive frameworks, which appear promising as future extensions of this work, in particular through adaptive dynamic variants of DPCA.

Moreover, Jorry et al. [23] developed a hybrid strategy combining MSPC with Fourier transforms and genetic algorithms for bearing failure detection, achieving high diagnostic accuracy through time-domain indicators. The authors emphasise the relevance of robust multivariate schemes for rotating machinery monitoring.

From another condition monitoring perspective, the literature shows that, in rotating systems with variable speed, non-stationary operation, or high sensor complexity, strategies such as angular resampling, NOC references based on spectral kurtosis, multi-source fusion, and hybrid approaches based on time–frequency representations and convolutional neural networks can improve diagnostic stability and failure identification [52–56]. Although these mechanisms are not part of the methodological workflow adopted in this study, their findings help to contextualise the challenges associated with operating variability and reinforce the relevance of the proposed DPCA–MSPC scheme; within this framework, MSPC retains its role in early warning and statistical interpretability, while subsequent supervised approaches may be considered complementary tools for discriminating failure types and severity levels.

Finally, the available evidence supports the suitability of the approach adopted in this study, which extends the application of DPCA–MSPC to high-criticality mechanical components such as spur gearboxes. The combination of diagnostic sensitivity, temporal modelling, and computational efficiency positions this methodology as a robust tool for continuous monitoring and decision-making in predictive maintenance under demanding industrial conditions. Therefore, implementing DPCA within MSPC frameworks constitutes a substantive contribution to the development of advanced data-driven diagnostic strategies.

## 5 Conclusions

This study demonstrates that integrating DPCA within an MSPC framework provides an effective, interpretable, and computationally efficient strategy for early failure detection in spur gearboxes. The proposed scheme calibrates model complexity via time-block CV, minimising out-of-sample *SPE* under a controlled *ARL*_0_ constraint, and then freezes the Phase I statistical limits for use in Phase II. For the analysed dataset, the selected configuration was (*p*,*k*)=(0,8) with α=0.01, applying fixed ${UCL}_{T^{2}}$ and *UCL_SPE_* across all failure severities (Table 3).

Combining Hotelling’s *T*^2^ (principal subspace) and *SPE* (residual space) provides a complementary and robust characterisation of deviations from NOC. Consistent with the methodological protocol, in Phase II the *SPE* chart systematically triggers alarms earlier than *T*^2^ for all severities (${\tilde{d}}_{SPE}\;\approx \;16.5$ versus ${\tilde{d}}_{T^{2}}\;\approx \;21$ segments), and the post-onset maxima exhibit a weak but statistically significant monotonic association with severity for *SPE* (Spearman $\hat{\rho }\;\approx \;-0.10$, *p* < 0.01), but not for *T*^2^ ($\hat{\rho }\;\approx \;0.02$, *p* = 0.66). This asymmetry is consistent with a physical–statistical mechanism in which early damage manifestations first perturb the correlation structure learned under NOC, injecting energy into the residual space (*SPE*) before inducing sustained shifts within the principal subspace (*T*^2^).

From a methodological perspective, this work extends the DPCA–MSPC framework to spur gearboxes, which are critical components in many mechanical systems, through an unsupervised, data-driven calibration that does not require labelled failures. Freezing the Phase I limits ensures objective and comparable decisions in Phase II, while jointly using *T*^2^ and *SPE* increases sensitivity to shifts in the principal subspace and to changes in the residual structure. Unlike most previous studies focused on bearings or wind turbines, and to the best of our knowledge, this is the first documented application of an DPCA–MSPC scheme to spur gearbox diagnosis, bridging engineering-oriented vibration analysis and data-driven statistical process control.

Validation was conducted under controlled laboratory conditions, which may limit extrapolation to plant environments with greater variability and noise. Although the test bench provided additional signals (acoustic emission, airborne sound, voltage/current), we deliberately chose not to integrate multi-signal analysis in order to isolate the specific contribution of vibration under an DPCA–MSPC protocol with frozen thresholds.

Future work will proceed in three directions: (i) incorporating adaptive schemes for non-stationary conditions with online parameter adjustment while maintaining a controlled *ARL*_0_; (ii) validating the framework in plant settings under variable operating regimes and real disturbances; and (iii) exploring signal fusion and adaptive variants of DPCA to strengthen sensitivity to incipient degradation and improve cross-domain transferability.

## Supporting information

S1 Appendix A: Condition Indicators, B: Illustration of the DPCA-MSPC framework (Pipeline), and C: Figures should be included. (ZIP)
