# Supplementary material for: Early fault detection in gearboxes via dynamic principal component analysis–driven multivariate statistical process control
Source: PLoS One. 2026 May 18;21(5):e0348497. doi: 10.1371/journal.pone.0348497 (PMC13183289; doi:10.1371/journal.pone.0348497)
Supplement: S1 Appendix — (ZIP) [file pone.0348497.s001.zip › S1 Appendix.docx]

Appendix

A Condition indicators

Table 7. **Condition indicators in time and frequency domains**

| **Condition indicator** | **Formula** | **Description** |
| --- | --- | --- |
| **Time domain** | | |
| Mean | <<Eqn136>> | Mean value of the acceleration signal. |
| Standard deviation | <<Eqn137>> | Dispersion of values with respect to the mean. |
| Kurtosis | <<Eqn138>> | Peakedness (tail heaviness) of the distribution. |
| Skewness | <<Eqn139>> | Asymmetry of the signal. |
| Shape factor | <<Eqn140>> | Ratio between Root Mean Square (RMS) and absolute mean value. |
| Impulse factor | <<Eqn141>> | Ratio between the peak value and the absolute mean value. |
| Clearance factor | <<Eqn142>> | Sensitivity to impulsive content (clearance in amplitudes). |
| Crest factor | <<Eqn143>> | Ratio between peak value and RMS. |
| Zero crossing | <<Eqn144>> | Number of times the signal crosses zero axis. |
| Higher-order temporal moment | <<Eqn145>> | Higher-order moments (default *m*=3). |
| **Frequency domain** | | |
| Spectral skewness | <<Eqn146>> | Asymmetry of the power spectrum relative to the spectral mean <<Eqn147>>. |
| Spectral kurtosis | <<Eqn148>> | Concentration (peakedness) of spectral energy. |
| Central frequency | <<Eqn149>> | Central frequency of the viobration spectrum. |
| Spectral standard deviation | <<Eqn150>> | Spectral deviation relative to the central frequency *F*_3_. |
| Spectral RMS | <<Eqn151>> | Overall spectral energy (global vibration magnitude). |
| Relative dispersion ratio | <<Eqn152>> | Dispersion relative to the central frequency *F*_3_. |
| Shape indicator | <<Eqn153>> | Ratio between fourth and second order spectral moments. |
| Second spectral moment | <<Eqn154>> | Dispersion of spectral energy with respect to squared frequency. |
| Third spectral moment | <<Eqn155>> | Spectral tilt toward higher or lower frequencies. |
| Fourth spectral moment | <<Eqn156>> | Sharpness or concentration of spectral peaks. |

*N* is the number of time samples <<Eqn157>>; <<Eqn158>>. For spectral terms, *X*(*k*) denotes the spectrum amplitude or power at bin *k* with center frequency <<Eqn159>>; when *P*(*k*) appears, it refers to a normalized spectral density. <<Eqn160>> and <<Eqn161>> denote the mean and variance across frequency bins, respectively.

B Illustration of the DPCA–MSPC framework (Pipeline)

This appendix presents a sequential and reproducible implementation of the proposed DPCA–MSPC framework using real vibration data obtained from the test bench described in Section 3. The aim is to clearly demonstrate how the procedure is applied in practice, following the classical stages of statistical process control: exploratory data analysis, Phase I model calibration using data under normal operating conditions, and Phase II monitoring under failure conditions.

Exploratory data analysis

Before calibrating the monitoring model, we conducted an exploratory analysis of the segmented vibration features extracted from the dataset corresponding to the normal operating condition (*P*_0_). This analysis had a dual purpose: first, to characterise the distribution, variability, and correlation structure of the condition indicators extracted at the segment level; and second, to verify the presence of temporal dependence between consecutive segments. This stage is essential, as it justifies both variable standardisation and extending the classical PCA model to a dynamic approach (DPCA) when relevant autocorrelation is detected.

Each segment is defined as a non-overlapping block of consecutive vibration samples of length *b*, from which a multivariate feature vector is extracted. Therefore, each segment constitutes the basic observational unit for the MSPC model. At this stage, we visualise the signal in the time domain, obtain its spectral representation, segment the signals, and compute descriptive statistics, the correlation matrix, and the autocorrelation functions of the extracted features.

**Algorithm B1** Data preparation, descriptive analysis, and dynamic structure assessment

**Require:** Raw HDF5/.mat signals; dataset path; channel index; sampling frequency <<Eqn162>>; block size *b*

**Ensure:** Descriptive statistics for in-control data and feature representations for subsequent monitoring stages

1: Read the vibration signal from the selected channel

2: Visualize the signal in the time domain and compute its frequency spectrum

3: Partition each signal into non-overlapping segments of length *b*

4: **for** each segment *t* **do**

5: Extract the feature vector <<Eqn163>>

6: **end for**

8: Construct the segment-level dataset <<Eqn164>>

9: Compute descriptive statistics of each feature under *P*_0_:

<<Eqn165>>

9: Inspect feature variability and scale differences across segments

10: Estimate the feature correlation matrix and assess linear dependence among indicators

11: **for** each feature series in <<Eqn166>> **do**

12: Compute the autocorrelation function (ACF) across ordered segments

13: **end for**

14: Assess the presence of temporal dependence in the segmented observations

15: **if** temporal correlation is detected **then**

16: Motivate the inclusion of lagged observations

17: Select DPCA as the monitoring model

18: **else**

19: Retain static PCA as baseline

20: **end if**

21: Define each segment as the basic observation unit for MSPC

22: Assign *P*_0_ as Phase I (in-control reference data)

23: Assign <<Eqn167>> as Phase II (fault evaluation data)

24: **return** Descriptive statistics table, feature vectors <<Eqn168>>, and dataset <<Eqn169>> for Phase I and Phase II

Algorithm B1 yields the descriptive statistics of the reference dataset under normal operating conditions, which are summarised in Table 2 in Section 4. In addition, this algorithm generates the initial visualisation of the signal in the time and frequency domains, shown in Fig. 3 in Section 3.

It is important to note that this algorithm does not yet produce control limits, alarm decisions, or detection delays. Its role is to generate the inputs required for subsequent stages, including segment-level feature vectors and the data matrix used for Phase I calibration and Phase II monitoring. Methodologically, this stage serves as exploratory data analysis within the classical MSPC scheme.

Phase I: model calibration under normal operating conditions

Once the exploratory analysis has been completed, we proceed to calibrate the model using the reference data under normal operating conditions (*P*_0_). In this phase, we estimate the standardisation parameters, select the hyperparameters of the dynamic model, fit the DPCA subspace, and compute the control limits. All these elements are subsequently frozen for use in Phase II, without any readjustment when failure data are introduced.

**Algorithm B2** Phase I: calibration and freezing

**Require:** Dataset *P*_0_; grid <<Eqn170>> for (*p*,*k*); significance level <<Eqn171>>; target *ARL*_0_; time-blocked cross-validation scheme

**Ensure:** Frozen parameter set for Phase II monitoring

1: Estimate <<Eqn172>> from *P*_0_ and standardize the dataset

2: **for all** <<Eqn173>> **do**

3: Define time-blocked folds to prevent temporal leakage

4: **for all** folds **do**

5: Construct the DPCA data matrix <<Eqn174>> using lagged vectors <<Eqn175>>

6: Fit DPCA on training blocks <<Eqn176>>

7: Compute *SPE* on validation blocks

8: **end for**

9: <<Eqn177>> average validation *SPE* across folds

10: Estimate *ARL*_0_(*p*,*k*) under NOC via simulation or resampling at level <<Eqn178>>

11: **end for**

12: Select <<Eqn179>> by minimizing validation *SPE* under an *ARL*_0_ constraint

13: Refit DPCA on the full standardized *P*_0_ using <<Eqn180>> to obtain <<Eqn181>> and <<Eqn182>>

14: Compute control limits at level <<Eqn183>>:

<<Eqn184>> using the effective sample size *n*_0_

<<Eqn185>> using the residual eigenvalues

15: **Freeze and store**

<<Eqn186>>

16: **return** Frozen parameter set for Phase II

Table 3 is obtained from the final stages of Algorithm B2. This information is used to select the optimal model configuration, compute the control limits, and freeze the Phase I parameters. In other words, this table summarises the direct outcome of the calibration process: hyperparameter selection, estimation of the DPCA subspace, and definition of the statistical thresholds that will subsequently be used in Phase II.

Phase I uses only information from *P*_0_. In this way, any alarm signal observed later in Phase II can be interpreted strictly as a deviation from normal operating conditions, rather than as a consequence of model recalibration.

Phase II: monitoring under failure conditions

In Phase II, once the model has been calibrated and frozen, it is applied to real data corresponding to failure conditions (<<Eqn187>>). In this phase, we do not re-estimate the scaling parameters, the DPCA subspace, or the control limits. Each new segmented observation is standardised using the Phase I parameters, projected onto the frozen subspace, and evaluated using the *T*^2^ and *SPE* statistics, whose trajectories enable the detection, interpretation, and quantification of emerging anomalies.

**Algorithm B3** Phase II: monitoring workflow

**Require:** Frozen <<Eqn188>>

**Ensure:** Alarm indicators, stored monitoring statistics, and outputs for performance evaluation

1: Receive a new signal segment represented by its condition indicators at time *t*

2: **if** <<Eqn189>> **then**

3: Skip evaluation until enough past segments are available

4: **return** No decision

5: **end if**

6: Standardize the new observation using Phase I reference values:

<<Eqn190>>

7: Construct the dynamic observation vector:

<<Eqn191>>

8: Project <<Eqn192>> onto the frozen DPCA subspace:

<<Eqn193>>

9: Compute monitoring statistics:

<<Eqn194>>

<<Eqn195>>

10: Compare the statistics with the frozen control limits:

<<Eqn196>> if <<Eqn197>>, else <<Eqn198>>

<<Eqn199>> if <<Eqn200>>, else <<Eqn201>>

11: Optionally update EWMA/CUSUM charts for enhanced detection of gradual changes

12: Store *T*^2^(*t*), *SPE*(*t*), <<Eqn202>>, and <<Eqn203>> for detection-delay and performance analysis

13: **return** <<Eqn204>>

Algorithm B3 produces the main outputs of the monitoring stage. In particular, the temporal trajectories of *T*^2^ and *SPE* are used to construct Fig. 4 and Figs. 6–15, whereas the aggregated metrics derived from the first exceedances of the control limits are summarised in Table 4. In addition, descriptive statistics of the monitoring signals, including means, maxima, and percentages of out-of-control observations, are synthesised in Table 5.

This stage explicitly shows how a real failure signal is processed using a model fully frozen from Phase I, and how the first exceedance of the control limit defines the detection delay in segment units.

Comparison between PCA and DPCA

Algorithm B4 assesses the benefit of incorporating temporal information into the monitoring framework by comparing the static PCA model with the proposed DPCA approach, while keeping the same experimental signals, the same calibration scheme, and the same monitoring workflow, varying only the structure of the model used.

**Algorithm B4** Comparison between PCA and DPCA monitoring models

**Require:** Reference dataset *P*_0_; monitoring datasets <<Eqn205>>; significance level <<Eqn206>>

**Ensure:** Comparative monitoring statistics and visualization outputs

1: Define candidate monitoring models <<Eqn207>>

2: **for all** models *M* **do**

3: Apply Phase I calibration (Algorithm B2) on *P*_0_ using model *M*

4: Obtain frozen parameters:

<<Eqn208>>

5: **for all** datasets <<Eqn209>>, <<Eqn210>> **do**

6: Apply Phase II monitoring (Algorithm B3)

7: Record sequences *T*^2^(*t*) and *SPE*(*t*)

8: Count exceedances of <<Eqn211>> and <<Eqn212>>

9: Compute percentage of out-of-control observations for each statistic

10: **end for**

11: Compute performance metrics:

in-control exceedance rate in *P*_0_

run-length statistics (ARL, SDRL)

probability of alarm for *T*^2^ and *SPE*

detection delay statistics by severity

monotonicity assessment via Spearman rank correlation

12: **end for**

13: Compare performance metrics across PCA and DPCA models

14: Summarize numerical results in Table 6

15: Generate comparative visualization of exceedance percentages across severity levels (Fig. 5)

16: **return** Comparative monitoring statistics and visualization summaries

As a result of this procedure, Table 6 summarises the main comparative indicators of in-control stability and signalling capability obtained for PCA and DPCA, whereas Fig. 5 shows the evolution of the percentages of out-of-control observations across the different severity levels. Taken together, this comparison enables a direct assessment of the effect of incorporating temporal information within the MSPC scheme and supports the interpretation of the obtained results.

C Figures

Fig 6. *P*_0_ severity level: Visual comparison of Hotelling’s *T*^2^ statistics and *SPE*. The red line represents the control thresholds.

Fig 7. *P*_1_ severity level: Visual comparison of Hotelling’s *T*^2^ statistics and *SPE*. The red line represents the control thresholds.

Fig 8. *P*_2_ severity level: Visual comparison of Hotelling’s *T*^2^ statistics and *SPE*. The red line represents the control thresholds.

Fig 9. *P*_3_ severity level: Visual comparison of Hotelling’s *T*^2^ statistics and *SPE*. The red line represents the control thresholds.

Fig 10. *P*_4_ severity level: Visual comparison of Hotelling’s *T*^2^ statistics and *SPE*. The red line represents the control thresholds.

Fig 11. *P*_5_ severity level: Visual comparison of Hotelling’s *T*^2^ statistics and *SPE*. The red line represents the control thresholds.

Fig 12. *P*_6_ severity level: Visual comparison of Hotelling’s *T*^2^ statistics and *SPE*. The red line represents the control thresholds.

Fig 13. *P*_7_ severity level: Visual comparison of Hotelling’s *T*^2^ statistics and *SPE*. The red line represents the control thresholds.

Fig 14. *P*_8_ severity level: Visual comparison of Hotelling’s *T*^2^ statistics and *SPE*. The red line represents the control thresholds.

Fig 15. *P*_9_ severity level: Visual comparison of Hotelling’s *T*^2^ statistics and *SPE*. The red line represents the control thresholds.
