# Supplementary figures and images for: Early fault detection in gearboxes via dynamic principal component analysis–driven multivariate statistical process control
Source: PLoS One. 2026 May 18;21(5):e0348497. doi: 10.1371/journal.pone.0348497 (PMC13183289; doi:10.1371/journal.pone.0348497)

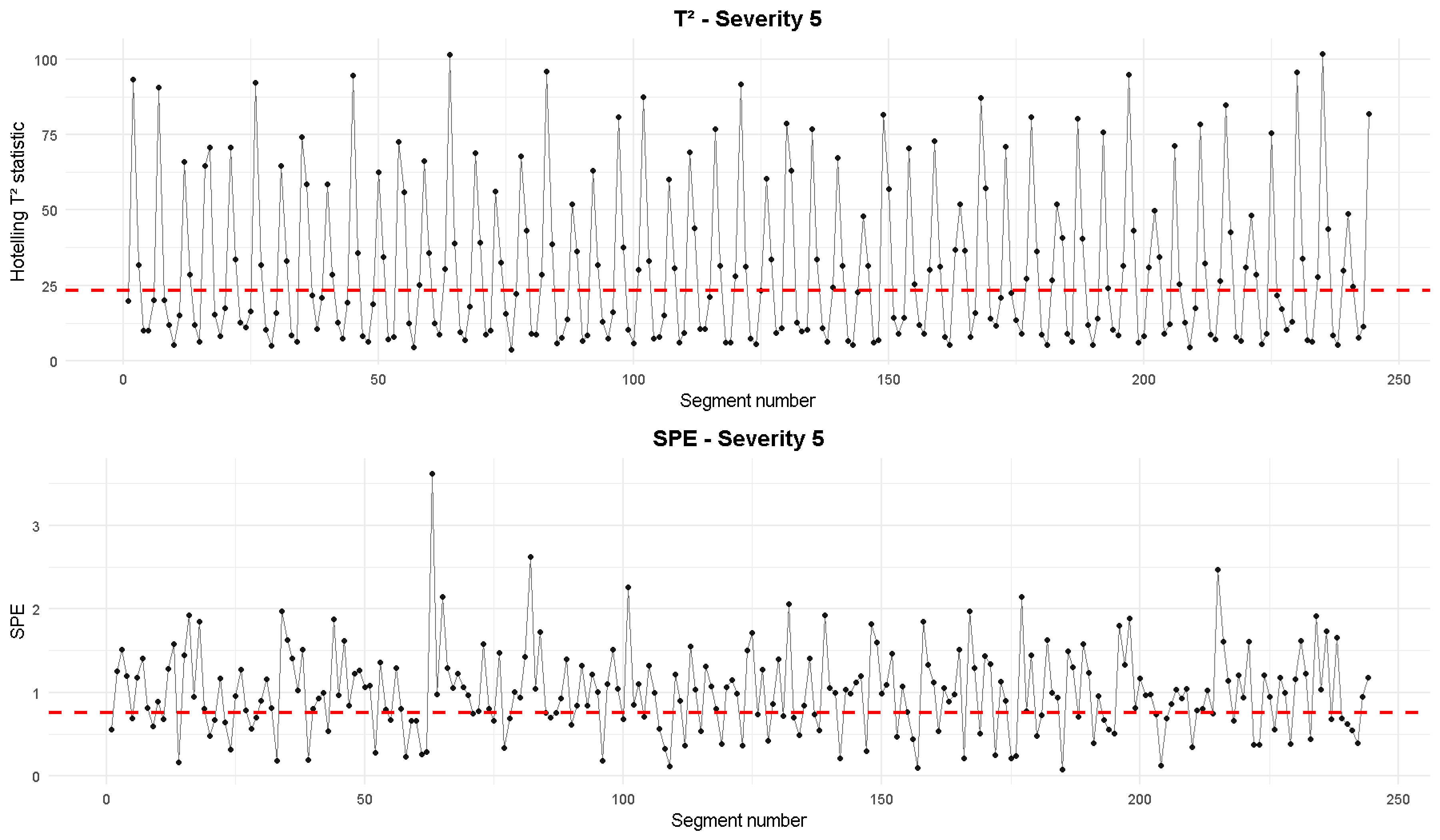

Supplement: S1 Appendix — (ZIP) [file pone.0348497.s001.zip › Fig 10.tif]

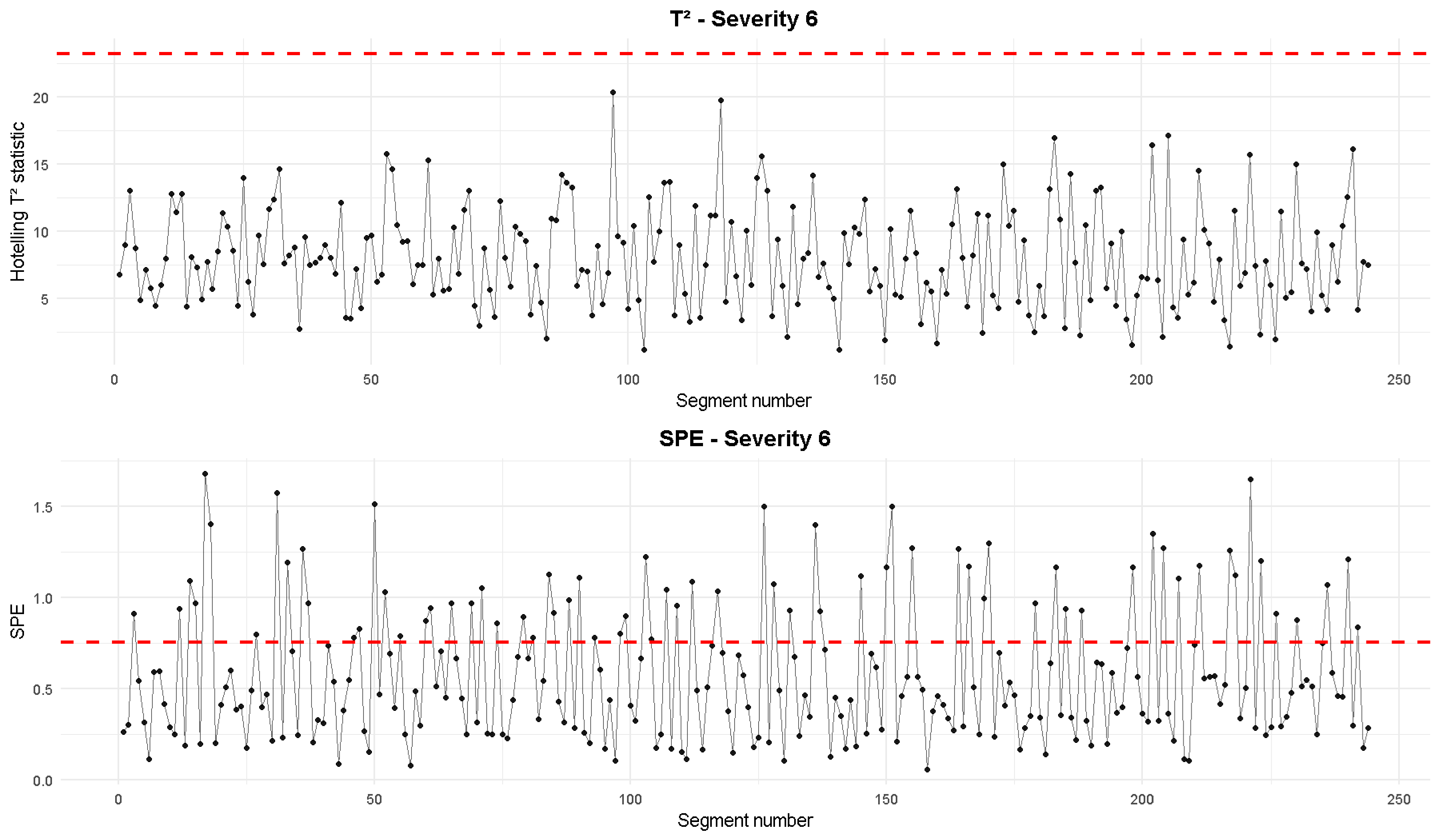

Supplement: S1 Appendix — (ZIP) [file pone.0348497.s001.zip › Fig 11.tif]

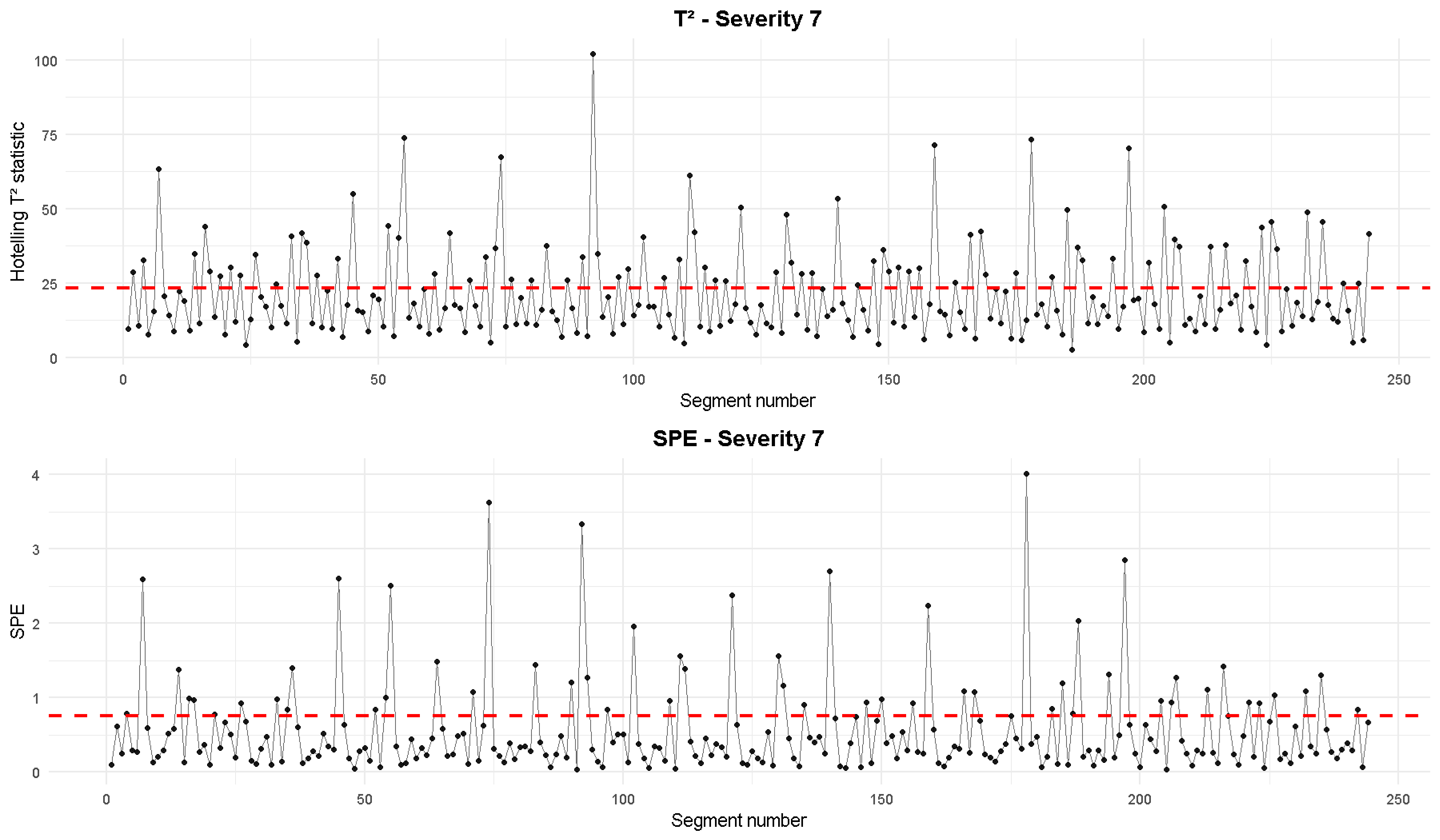

Supplement: S1 Appendix — (ZIP) [file pone.0348497.s001.zip › Fig 12.tif]

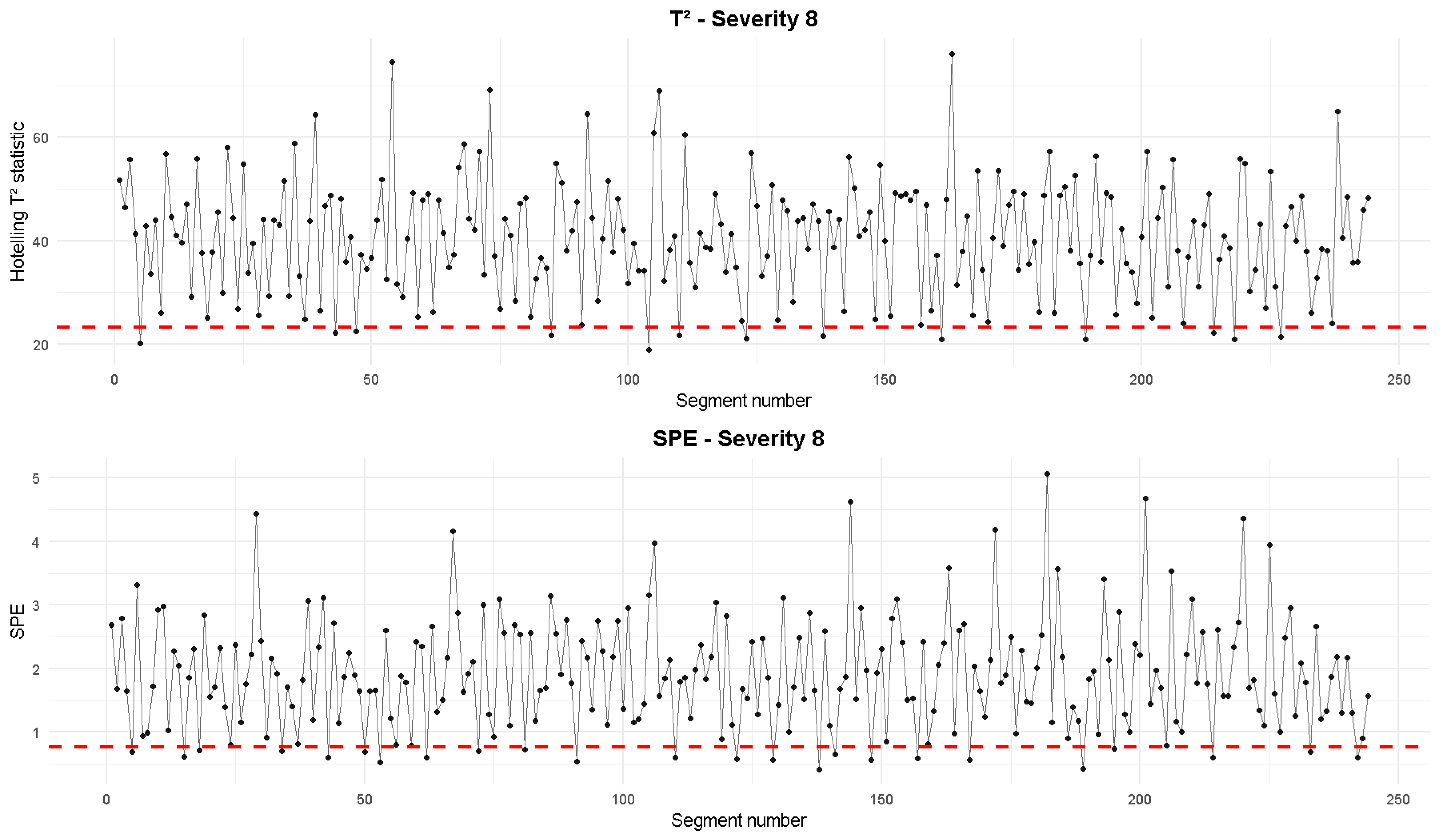

Supplement: S1 Appendix — (ZIP) [file pone.0348497.s001.zip › Fig 13.tif]

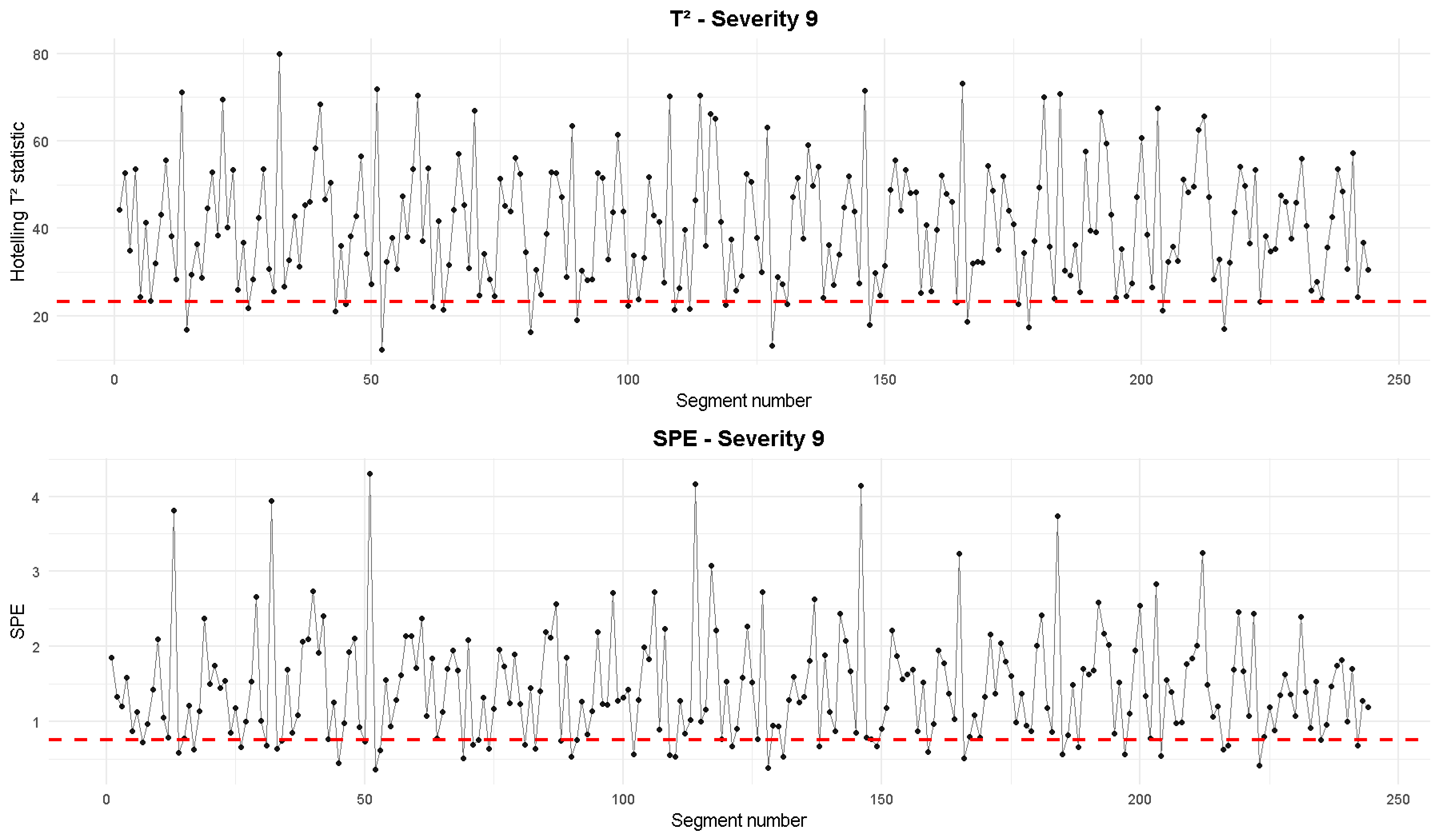

Supplement: S1 Appendix — (ZIP) [file pone.0348497.s001.zip › Fig 14.tif]

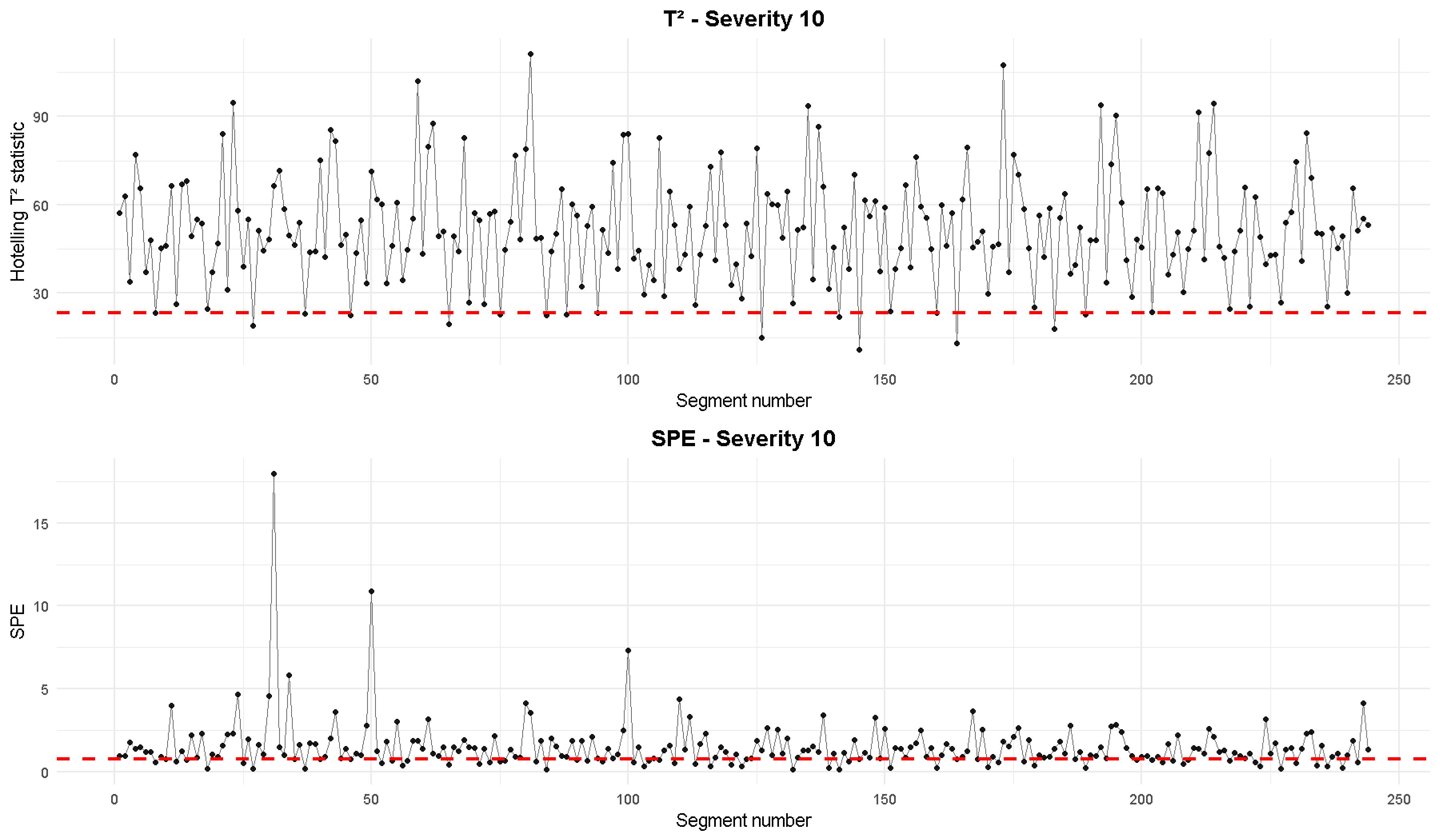

Supplement: S1 Appendix — (ZIP) [file pone.0348497.s001.zip › Fig 15.tif]

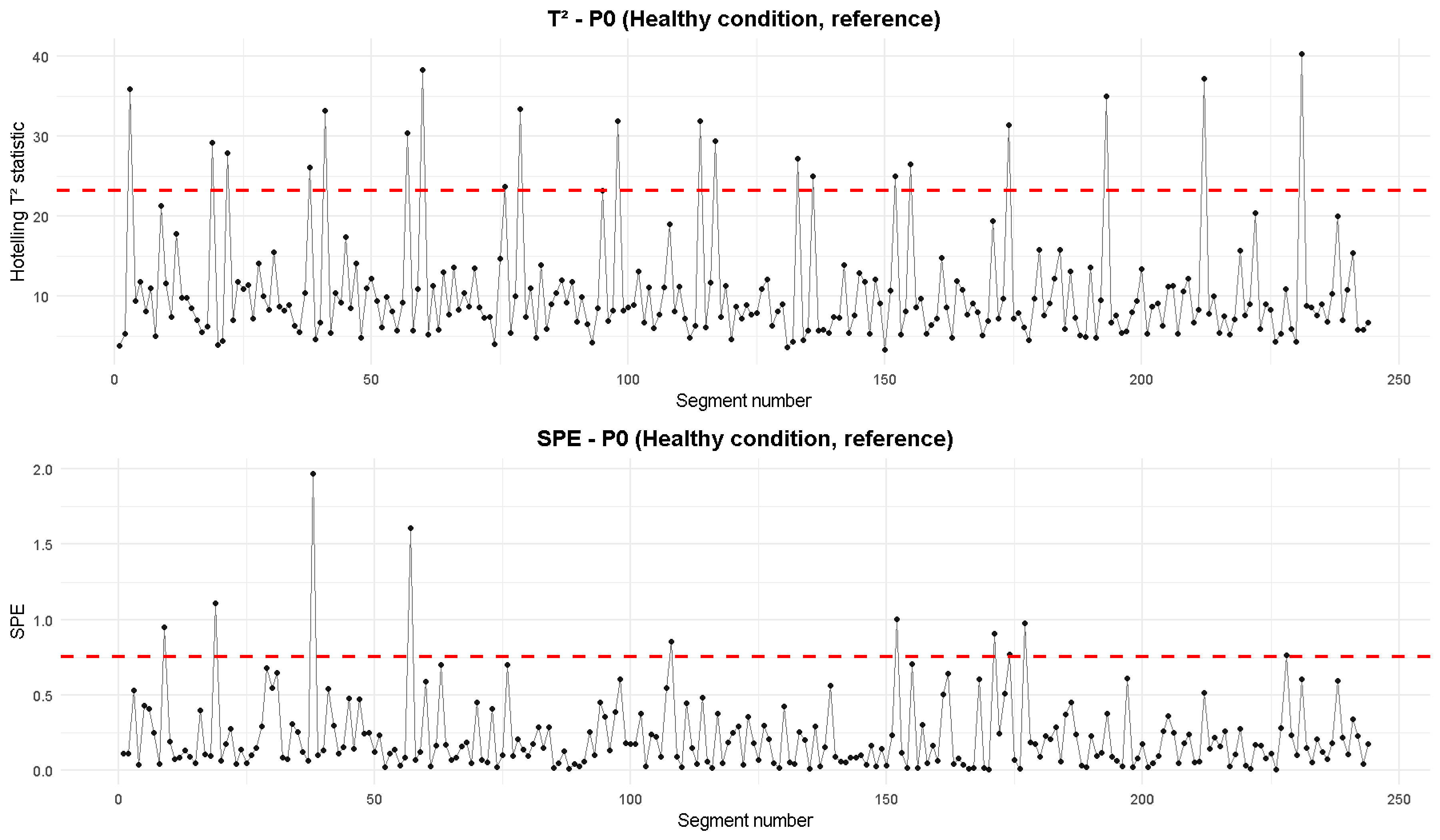

Supplement: S1 Appendix — (ZIP) [file pone.0348497.s001.zip › Fig 6.tif]

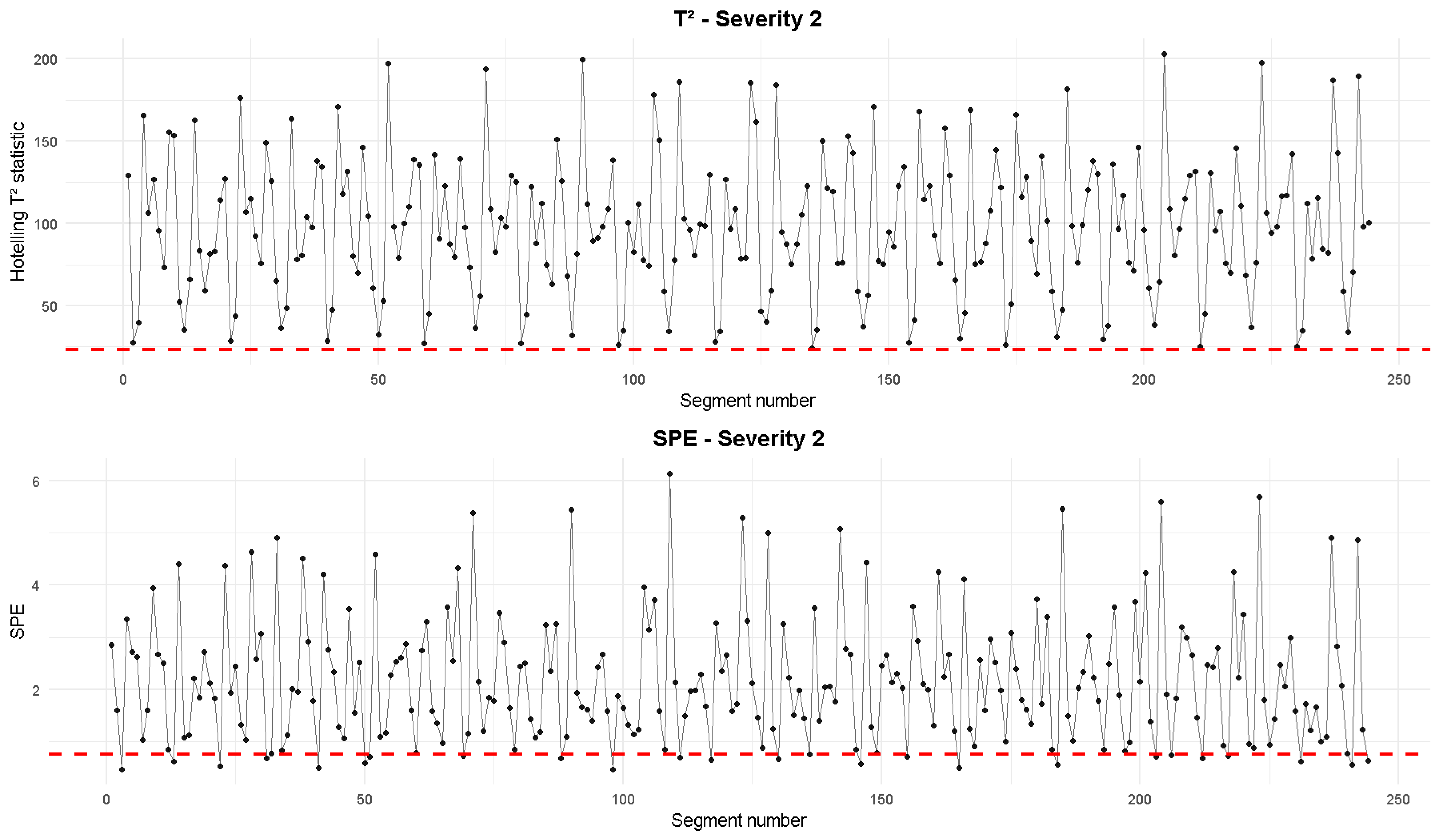

Supplement: S1 Appendix — (ZIP) [file pone.0348497.s001.zip › Fig 7.tif]

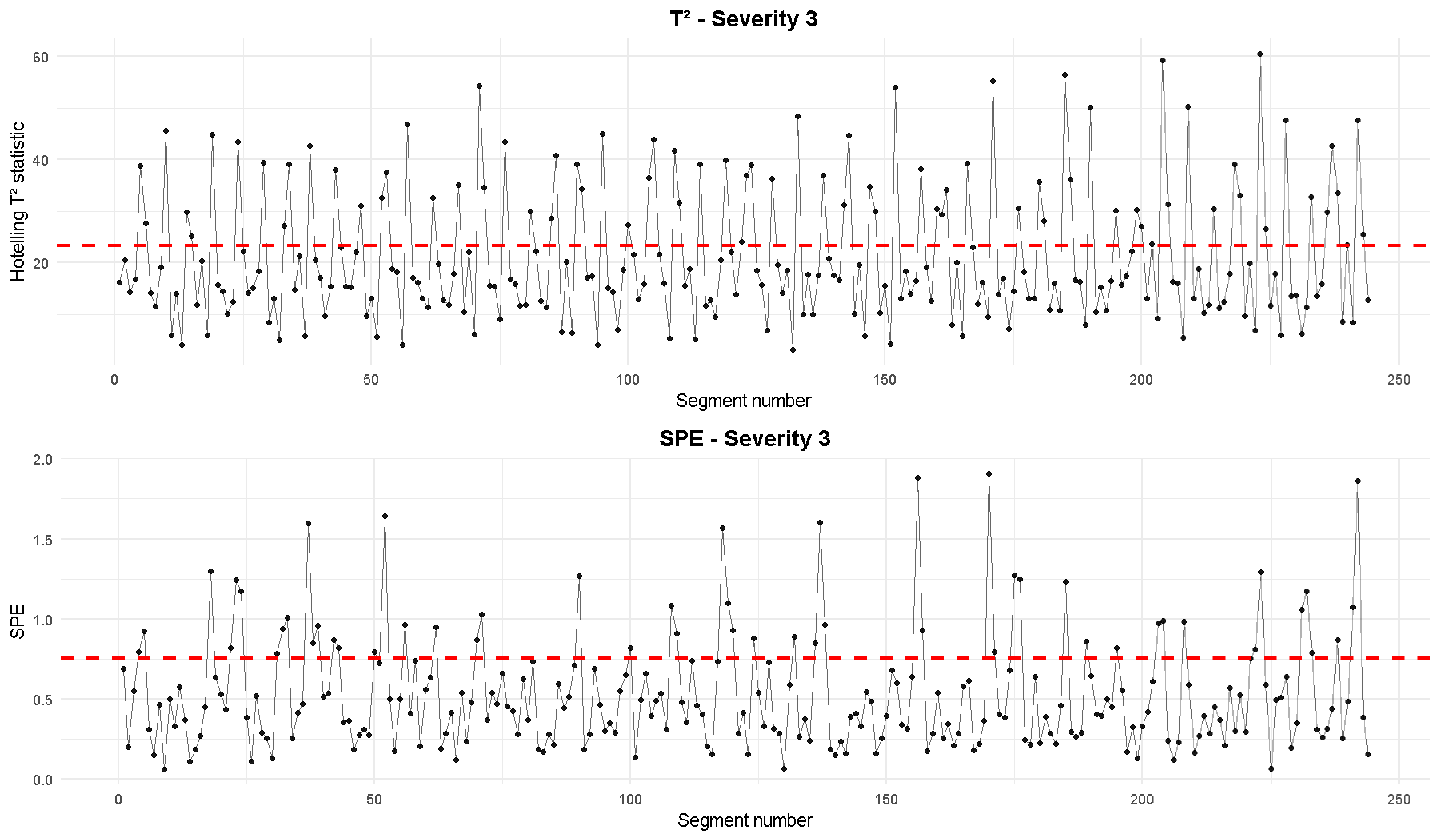

Supplement: S1 Appendix — (ZIP) [file pone.0348497.s001.zip › FIg 8.tif]

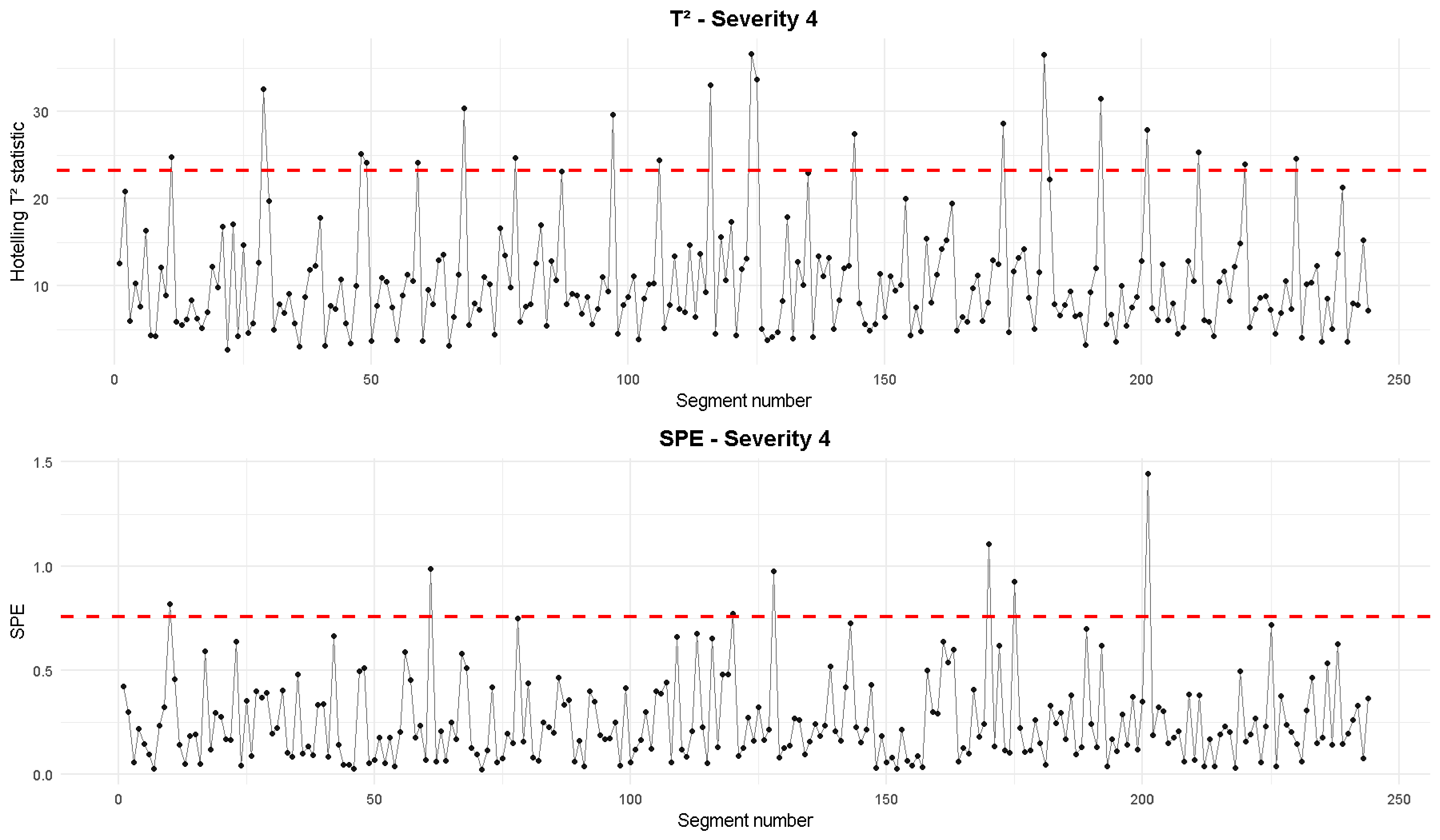

Supplement: S1 Appendix — (ZIP) [file pone.0348497.s001.zip › FIg 9.tif]
